# Energy depletion and opportunistic microbial colonisation in white syndrome lesions from corals across the Indo-Pacific

**DOI:** 10.1038/s41598-020-76792-x

**Published:** 2020-11-17

**Authors:** Hillary A. Smith, Jessica A. Conlan, F. Joseph Pollock, Naohisa Wada, Amanda Shore, Julia Yun-Hsuan Hung, Greta S. Aeby, Bette L. Willis, David S. Francis, David G. Bourne

**Affiliations:** 1 College of Science and Engineering, James Cook University, Townsville, QLD 4811 Australia; 2 School of Life and Environmental Sciences, Deakin University, Geelong, VIC 3216 Australia; 3 Penn State University, Eberly College of Science, 517 Thomas Building, University Park, PA 16802 USA; 4 Hawaii & Palmyra Programs, The Nature Conservancy, Honolulu, HI 96817 USA; 5 Biodiversity Research Center, Academia Sinica, Nangang, Taipei, 11529 Taiwan; 6 Department of BioSciences, Rice University, Houston, TX 77005 USA; 7 ARC Centre of Excellence for Coral Reef Studies, James Cook University, Townsville, QLD 4811 Australia; 8 Department of Biological and Environmental Sciences, Qatar University, Doha, Qatar; 9 AIMS@JCU, Australian Institute of Marine Science and James Cook University, Townsville, QLD 4811 Australia; 10 Australian Institute of Marine Science, Townsville, QLD 4810 Australia

**Keywords:** Microbiome, Metabolomics, Ecological epidemiology

## Abstract

Corals are dependent upon lipids as energy reserves to mount a metabolic response to biotic and abiotic challenges. This study profiled lipids, fatty acids, and microbial communities of healthy and white syndrome (WS) diseased colonies of *Acropora hyacinthus* sampled from reefs in Western Australia, the Great Barrier Reef, and Palmyra Atoll. Total lipid levels varied significantly among locations, though a consistent stepwise decrease from healthy tissues from healthy colonies (HH) to healthy tissue on WS-diseased colonies (HD; i.e. preceding the lesion boundary) to diseased tissue on diseased colonies (DD; i.e. lesion front) was observed, demonstrating a reduction in energy reserves. Lipids in HH tissues were comprised of high energy lipid classes, while HD and DD tissues contained greater proportions of structural lipids. Bacterial profiling through 16S rRNA gene sequencing and histology showed no bacterial taxa linked to WS causation. However, the relative abundance of Rhodobacteraceae-affiliated sequences increased in DD tissues, suggesting opportunistic proliferation of these taxa. While the cause of WS remains inconclusive, this study demonstrates that the lipid profiles of HD tissues was more similar to DD tissues than to HH tissues, reflecting a colony-wide systemic effect and provides insight into the metabolic immune response of WS-infected Indo-Pacific corals.

## Introduction

Coral reefs globally are under pressure from both local anthropogenic impacts and global climate factors^1,2^. These cumulative stressors are linked with increasing disease outbreaks that contribute to coral cover decline^3–7^. White syndromes (WSs) are a macroscopic grouping of prevalent coral diseases based on gross lesion characteristics that are reported across the Indo-Pacific, particularly affecting the dominant reef-forming family Acroporidae^8–11^. Visually, WSs manifest as a distinct lesion forming between affected and unaffected tissues. While coral tissue ahead of the lesion appears healthy, tissue at the lesion boundary is necrotic and actively sloughing away, revealing the bare white skeleton beneath^8,12,13^. These lesions can progressively migrate across a coral colony, resulting in either partial or whole colony mortality^8,14^.

The underlying factors leading to the onset of WS disease lesions are unknown, although it is likely that multiple modes of pathogenesis manifest similarly as slow or rapid tissue loss in corals. Hence, like many coral diseases, a number of biotic and abiotic factors are linked with WSs^8,10,15^. For example, complex synergistic effects between environmental and host factors contribute to disease onset, with outbreaks often correlated with warm seawater anomalies and high coral density^16–18^. In addition, biological agents including vibrios^19^, ciliates^20,21^, viruses^22^, parasites, and helminths^23^, as well as cellular apoptosis^24^ have been linked with WS disease causation. The presence of *Vibrio* *sp*. or other microbes suggests that such taxa may cause disease, or that they opportunistically proliferate in hosts with compromised health.

Corals have a suite of defences in their immune repertoire, including physical barriers (e.g. mucus, melanin deposits)^25–27^, molecular pattern recognition^28,29^, secretion of antimicrobial macromolecules^30^, and cellular and enzymatic responses (e.g. phagocytosis, prophenoloxidase, reactive oxygen species)^31–33^. When these defences fail and infection takes over, lesions and tissue mortality may manifest through molecular and cellular signatures of apoptosis^24,34,35^. For most coral species, the speed at which recovery occurs is critical to survival, since lesion progression rate is directly related to tissue mortality. As such, fast healing may preclude settlement and overgrowth by competing organisms or loss of physiological integration of the colony^12,36–38^. However, the upregulation of immunity is an energetically expensive process and the ability of corals to resist, respond and ultimately recover from disease-induced lesions is largely dependent on physiological traits that confer resilience, such as high energy reserves or beneficial microbial communities^39–41^.

Traditionally, total or ‘crude’ lipid concentration has served as a proxy for coral energy reserves, and is used to infer coral health status^42,43^. In the marine environment, lipids provide highly dense forms of energy, with around one-third more energy relative to proteins or carbohydrates^44^. Lipids are a major component of the coral proximate composition (10–40% of dry biomass) and their constituent classes and fatty acids provide important structural and energy storage functions^45,46^. Lipid stores in invertebrates are also involved in the regulation of innate immune homeostasis^47^. When a coral strays from homeostasis, its response is dependent on the colony’s physiological competence, thus total lipid content has been correlated with a coral’s ability to respond to stressors^48,49^. For example, large lipid stores can mitigate the detrimental effects of ocean warming and acidification^48,49^. Conversely, depletion of lipid reserves can increase susceptibility to disease and mortality^50,51^. Further, gene expression studies have shown that diseased coral tissues upregulate pathways associated with innate immunity, tissue repair, and lipid and carbohydrate metabolism, suggesting higher usage of stored lipids in diseased versus healthy coral tissue^31^.

Importantly, numerous experiments have shown that healthy corals preferentially direct energetic resources (e.g. metabolites and photoassimilates) toward physically-induced lesions for regeneration^12,31,52,53^. However, these same compounds are preferentially transferred away from disease-induced lesions^31^, including WS lesions^54^. This is consistent with the absence of tissue regeneration observed at degenerative WS lesion borders^18,54^. Assuming that corals possess finite energy reserves available for life functions^55,56^, the preferential translocation of energy reserves away from WS lesions may represent a ‘shutdown’ response of the colony to the rapid expansion of necrotic tissues to prevent further resource loss^54^, thereby protecting the remaining colony^57^. Examining lipid profiles between diseased and healthy tissues on the same coral colony would provide valuable insight into the transfer and partitioning of compounds around the colony as part of the overall immune response to WSs.

Coral-associated microorganisms including protozoa, fungi, bacteria, archaea, and viruses (collectively termed the microbiome) also contribute to host functioning and fitness^6,58,59^, and thus coral resilience is tightly linked with its associated microbial community. It is likely that the coral microbiome is involved in coral immunity either directly through production of antimicrobial compounds^60^ or indirectly through niche exclusion of opportunistic or pathogenic organisms^61,62^. While some microbial taxa are directly linked to disease onset^63^, it has also been proposed that changes to the coral host’s normal microbial community composition (i.e. dysbiosis) can induce disease or disease-like signs^63–65^. Host immune processes can also be involved in the establishment and maintenance of stable microbial communities during stress or infection^66,67^, thus potentially draining host resources such as lipid reserves. As such, comparison of microbial communities between healthy and diseased coral tissues can give further insight into the holobiont response to disease and may establish a link between coral physiology and its microbiome.

The present study profiled lipids, lipid classes, fatty acids, and the microbial communities of healthy tissue from colonies of healthy *Acropora hyacinthus* (HH), seemingly healthy tissue on WS-diseased colonies (HD; i.e. preceding the lesion boundary) and diseased tissue on WS-diseased colonies (DD; i.e. lesion front) from three locations across the Indo-Pacific: Western Australia (WA), the Great Barrier Reef (GBR), and Palmyra Atoll (PA). This work aimed to identify patterns that might indicate a holobiont-wide systemic effect of WSs, as well as providing insight into the immune response process. Microbial communities were also profiled to identify changes in holobiont community structure, with the patterns compared with previously published data from WS lesions from the Great Barrier Reef^68^.

## Methods

### Study site and sample collection

Samples for this study were collected from the Montebello and Barrow Islands, Western Australia (WA; 20°30′S, 115°31′E) in late Austral spring, December 2011 (n = 7 colonies; 4 diseased, 3 healthy) and Palmyra Atoll National Wildlife Refuge (PA; 5°53′N, 162°5′W) in the late Northern Hemisphere spring, May 2011 (n = 8 colonies; 4 diseased, 4 healthy). Palmyra Atoll was accessed under US Fish and Wildlife Service Permit 12533-11025. This sample set was supplemented for comparative analysis with samples from the study of Pollock et al.^68^ (n = 19 diseased, 21 healthy) derived from an 18-month sampling period at Lizard Island (GBR; 14º40′S, 145º27′E) in the northern sector of the GBR. Adult WS-affected colonies (i.e. 50–150 cm diameter colonies displaying diffuse, acute to sub-acute areas of tissue loss revealing white, intact skeleton) and nearby conspecific healthy control colonies of *Acropora hyacinthus* were identified on SCUBA (1–12 m depth). Fragments were collected from one location on each healthy colony and two locations on each WS-infected colony (i.e., one from the disease lesion interface and one from apparently healthy tissue approximately 10 cm away from the lesion). Within each colony, one fragment (~ 5 cm in length) was collected for bacterial community and energetics profiling and a second smaller fragment (~ 3 cm) was collected for histological analyses using surgical bone cutters. All colonies were tagged and photographed before and after sample collection. Within 15 min of collection, fragments for bacterial community and energetics profiling were snap-frozen in liquid nitrogen and stored at − 80 °C until processing. Fragments for histological analysis were placed in sterile, freshly prepared 4% paraformaldehyde (Electron Microscopy Sciences, USA), 10 mM phosphate buffered saline (PBS) solution at 4 °C. Samples from each site were divided into healthy tissues on healthy colonies (HH); healthy tissues on diseased colonies (HD); and disease lesion regions on diseased colonies (DD).

### Histological processing and fluorescence in situ hybridisation

Samples for histological analysis (n = 45) were processed as detailed previously^68,69^. Histological observations were recorded using a Leica DMI 6000B light microscope (Leica, Germany) and microphotograph images were processed using the LAS imaging software (Leica, Germany). Three sections from each sample were examined for signs of tissue response (i.e. necrosis, fragmentation, swelling) and foreign organisms (i.e. helminths, ciliates, fungi, cyanobacteria).

Samples from WA (n = 5) were used for fluorescence in situ hybridisation (FISH) to assess the distribution of bacteria in HH, HD, and DD tissues. DD tissues were further divided into three sections: D1 (where tissue was still intact), D2 (the lesion front; the interface between intact and compromised tissue), and D3 (where tissue integrity was clearly compromised, > 1 mm behind the lesion front). Samples were processed as previously described using EUB338 mix and VibGV probes targeting 16S rRNA gene and *Vibrio* species, respectively, and labelled with the Cy3 flurochrome (Thermo Fisher Scientific, Germany)^70,71^. An LSM710 confocal laser scanning microscope (Carl Zeiss, Germany) combined with spectral emissions profiling was used to visualize tissue-associated, FISH-labelled bacterial communities as described by Wada et al.^69^, and relative ratios of bacterial signal to coral tissue autofluorescence were quantified using a thresholding procedure^72^ in FIJI software (https://fiji.sc/wiki/index.php/Fiji).

### Total lipid and ash

Coral skeletons were crushed using a French press and the resultant coral powder was extracted for total lipid content as previously described^73^. Samples were weighed then soaked overnight in a 3 mL aliquot of 2:1 dichloromethane: methanol (CH_2_Cl_2_:CH_3_OH). The following morning, this mixture was filtered and the solid residue re-suspended and soaked for a further 10 min with another 3 mL aliquot of 2:1 CH_2_Cl_2_:CH_3_OH, followed by a further filtration step. This process was repeated three times. The combined filtrates (~ 9 mL) were then transferred into a separation funnel and combined with a 4.5 mL sample washing solution of KCl (0.44%) in H_2_O/CH_3_OH (3:1). The mixture was shaken vigorously and allowed to settle overnight. The following morning, the bottom layer containing the extracted lipid was recovered and the solvent was evaporated under nitrogen. The lipid content was then quantified on a 4-figure balance. Total ash was determined by incineration in a muffle furnace (C & L Fetlow, Model WIT, Blackburn, Victoria, Australia) at 450 °C for 12 h. The ash content was subtracted from the total composition to obtain ash free dry weight (AFDW), which excludes the inorganic component.

### Lipid class composition

A 1 μL aliquot of the total lipid fraction was taken and analysed for lipid class composition using an Iatroscan MK-6s thin layer chromatography—flame ionisation detector (Mitsubishi Chemical Medience, Tokyo, Japan) according to methods detailed previously^73^. Briefly, each sample was spotted in duplicate on silica gel S5-chromarods (5 μm particle size) and developed in a glass tank containing filter paper. Lipid separation followed a two-step elution sequence: 1) elution of phosphatidylcholine (PC), phosphatidylserine and phosphatidylinositol (PSPI), phosphatidylethanolamine (PE), and lysophosphatidylchloline (LPC) was achieved in a dichloromethane/methanol/water (50:20:2, by volume) solvent system run to half height (~ 15 min); and 2) after air drying, elution of wax esters (WAX), triacylglycerol (TAG), free fatty acid (FFA), sterol (STEROL), and 1,2-diacylglycerol (1,2-DAG) was achieved in a hexane/diethyl ether/formic acid (60:15:1.5, by volume) solvent system run to full height (30 min). Rods were then placed in an oven at 100 °C for 5 min prior to analysis. The Iatroscan MK-6s was calibrated using known compound classes in the range of 0.1–10 μg and peaks were quantified using PowerChrom version 2.7.13 (eDAQ Pty Ltd.). Given the presence of unknown, lipid-soluble pigments in the lipid fraction, the lipid class peaks arising between approx. 0.2–0.31 min were classified as “acetone mobile polar lipids” (AMPL)^44,74^, and will henceforth be referred to as such (these lipids were included in the structural lipid fraction due to the high amount of glycolipid).

### Fatty acid composition

Following extraction, fatty acids were esterified into methyl esters using the acid catalysed methylation method^75^. 100 μL of 23:0 (0.75 mg mL^−1^) was added as an internal standard (Sigma-Aldrich, Inc., St. Louis, MO, USA) alongside 2 mL of freshly prepared AcCl/ MetOH (1:10) as the methylation catalyst. Sample vials were then sealed, shaken and placed in an oven at 100 °C for 1 h. Once cool, 2 mL of K_2_CO_3_ (1 M) was added, followed by 3 mL of hexane to dissolve the fatty acid methyl esters. The sample was then centrifuged and the hexane supernatant recovered and placed in a gas chromatography (GC) vial for GC injection.

Fatty acid methyl esters were isolated and identified using an Agilent Technologies 7890A GC System (Agilent Technologies; Santa Clara, CA, USA) equipped with a BPX70 capillary column (120 m × 0.25 mm internal diameter, 0.25 μm film thickness, SGE Analytical Science, Ringwood, VIC, Australia), a flame ionization detector (FID), an Agilent Technologies 7693 auto sampler, and a splitless injection system. The injection volume was 1 μL and the injector and detector temperatures were 300 °C and 270 °C, respectively. The temperature program was 60 °C held for 2 min, then from 60 to 150 °C at 20 °C min^−1^, and held at 150 °C for 2 min, then from 150 to 205 °C at 1.5 °C min^−1^, then from 205 to 240 °C at 5 °C min^−1^, and held at 240 °C for 24 min. The carrier gas was helium at 1.5 mL min^−1^, at a constant flow. Each of the fatty acids was identified relative to known external standards (a series of mixed and individual standards from Sigma-Aldrich, Inc., St. Louis, MO, USA and from NuChek Prep Inc., Elysian, MN, USA), using the software GC ChemStation (Rev B.04.03; Agilent Technologies; Santa Clara, CA, USA). The resulting peaks were then corrected by the theoretical relative FID response factors^76^ and quantified relative to the internal standard.

### Lipid and fatty acid data statistical analysis

Statistical analysis for all lipid data was implemented in R software version 3.5.0^77,78^. Normality and heteroscedasticity were determined by performing the Shapiro–Wilk and Bartlett’s tests, respectively. Due to heteroscedasticity, differences between treatments were analysed using the non-parametric Kruskal–Wallis test. Where significant differences were detected, a t-student post hoc test was employed at a significance level of *p* < 0.05 to determine which groups differed (*agricolae* package^79^). Figures were prepared using the ggplot2 package^80^. Lipid class (% lipid) and fatty acid profiles (% fatty acid) were also analysed with one-way PERMANOVAs using 999 permutations. These analyses were visualized with a redundancy analysis (RDA) with 11 identified lipid classes visualized by overlaying loading vectors on the biplot. For fatty acids, a similarity of percentages (SIMPER) analysis was conducted with 999 permutations to determine the contribution of each fatty acid to between-group dissimilarity and the loading vectors of the top 15 fatty acids from the SIMPER analysis were displayed in the ordination plot. One outlier colony (paired HD and DD samples) was identified when compared across all other samples, and this colony was removed and analyses repeated.

### Bacterial 16S rRNA gene amplicon profiling and analysis

Extraction of total DNA from coral tissues and amplification of the V1–V3 region (*E. coli* position: 27–519) of the small-subunit ribosomal RNA (16S rRNA) gene was performed on all samples with forward primer 27F (GAGTTTGATCNTGGCTCAG) and reverse primer 519R (GTNTTACNGCGGCKGCTG), as described previously^81,82^. Amplicon sequencing was conducted as detailed in Pollock et al.^68^ and sequence reads were processed using QIIME2^83^. Briefly, samples were demultiplexed by sample-specific barcodes, reads were filtered for quality and chimeric sequences and dereplicated using the dada2^84^ plugin. Taxonomy was assigned using a naïve Bayes classifier trained on the extracted region of interest from the SILVA 16S rRNA reference database (release 132)^85^. Prior to downstream analysis, sequences classified as chloroplasts or mitochondria were removed. The resulting amplicon sequence variant (ASV) table at phylum, class, family, genus, and species levels were used for statistical analysis. All 16S rRNA gene sequences from the pyrosequencing analysis have been archived in the NCBI sequence read archive (SRA) under study accession number PRJNA524926.

Bacterial community alpha diversity (i.e. within sample diversity) was assessed by two-way ANOVA based on a linear model with Shannon’s diversity index as a response to the interaction between health state (levels: HH, HD, DD) and location (levels: GBR, Palmyra, WA). Beta diversity (i.e. between sample diversity) was assessed using a Bray–Curtis dissimilarity matrix based on Wisconsin-standardised count data at the ASV level. The dissimilarity matrix was used to construct non-parametric multidimensional scaling (nMDS) plots to visualize differences between bacterial community assemblages between health states and locations^86,87^. Following tests to ensure homogeneity of multivariate dispersion (BetaDisper), permutational multivariate analysis of variance (PERMANOVA^88^) was utilized to test for statistical differences in bacterial community assemblages between health states and locations. This analysis was based on a Bray–Curtis dissimilarity matrix, with 9999 permutations. Post hoc pair-wise comparisons among locations and health states were conducted following significant main effects results, and *p* values were adjusted for multiple comparisons using the Bonferroni correction.

Differentially abundant sequence variants between health states and locations were identified using correlation clustering as implemented by gneiss^89^ in QIIME2. Multivariate response linear regression was performed on the resulting balances based on health state and region, and balances with *p* values lower than 0.05 were further explored for taxonomy. All analyses were performed using R statistical software v3.5.0^77^ using the packages vegan^90^, ggplot2^80^, pheatmap^91^, pairwiseAdonis^92^, emmeans^93^, and multcompView^94^.

## Results

### Histological and FISH analysis of coral tissues

Tissues sampled from regions of healthy colonies (HH; n = 22) and healthy tissues from diseased colonies (HD; n = 23) histologically displayed no signs of necrosis, fragmentation or swelling, with the exception of one HH colony from the GBR that showed signs of swelling, and one HD sample from PA that showed signs of fragmentation in the healthy tissue region close to the lesion front. Helminths and fungi were observed in a small number (13%) of healthy tissue samples while ciliates and cyanobacteria were absent in all healthy tissues (HH and HD). In contrast, tissues from WS lesion fronts (DD; n = 23) displayed signs of necrosis (91% of samples) and fragmentation (74%). In addition, samples from the lesion front also displayed ciliates (17%), fungi (17%), cyanobacteria (13%), and helminths (9%; Supplementary Fig. S1).

FISH visualisation did not detect bacteria (total bacteria or vibrios) in HH (n = 2) or HD (n = 3) tissues from WA samples (Supplementary Fig. S2). Furthermore, no bacteria (except coral-associated microbial aggregates^95^) were observed in D1 regions (i.e. healthy tissue immediately adjacent to the lesion front (~ 1 mm) of DD samples; n = 3). At the WS lesion front (i.e. D2 region; interface of healthy and lesion tissue), bacteria and *Vibrio* were detected in all samples. Similarly, within diseased tissues (D3, > 1 mm behind the lesion front), bacteria were observed in all samples (though *Vibrio* were only observed in one sample).

### Total lipid concentration and class composition

**Figure 1:**
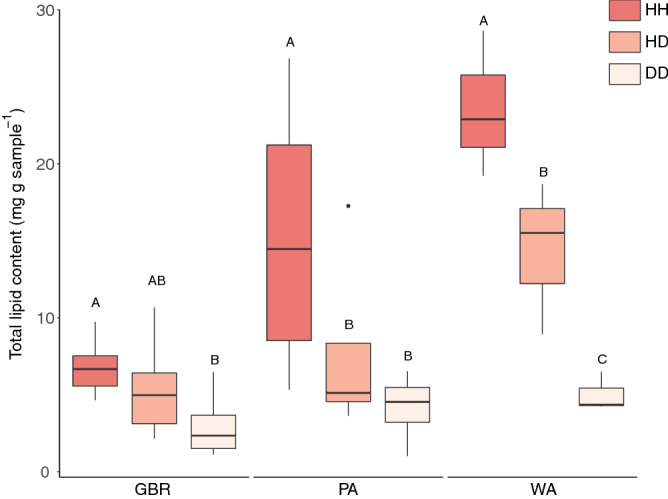
Mean total lipid content (mg g coral sample^−1^) in diseased and healthy colonies of *A. hyacinthus*. Letters above each boxplot indicate between health state (within each location) statistical significance. This figure was created in R Studio, version 1.2.5033 (https://rstudio.com).

For all sites, the healthy tissues (HH; n = 17) exhibited the highest total lipid concentrations (Fig. 1; Supplementary Table S1), which were significantly higher compared to diseased tissues (DD; n = 20; GBR: *p* = 0.041; WA: *p* < 0.0001; and PA: *p* = 0.0004)*.* Total lipids were also significantly higher in HH tissue than observed in HD tissues (n = 19), except in GBR samples (GBR: *p* = 0.55; WA: *p* = 0.012; PA: *p* = 0.019). Notably, there were marked differences in the lipid concentrations between regions (Supplementary Table S2). The highest concentrations were exhibited by WA, followed by PA, while the GBR samples contained the lowest concentrations across all health states. Indeed, the lowest total lipid concentration recorded for the WA samples was similar to the highest concentrations recorded at the GBR site (Fig. 1).

**Figure 2:**
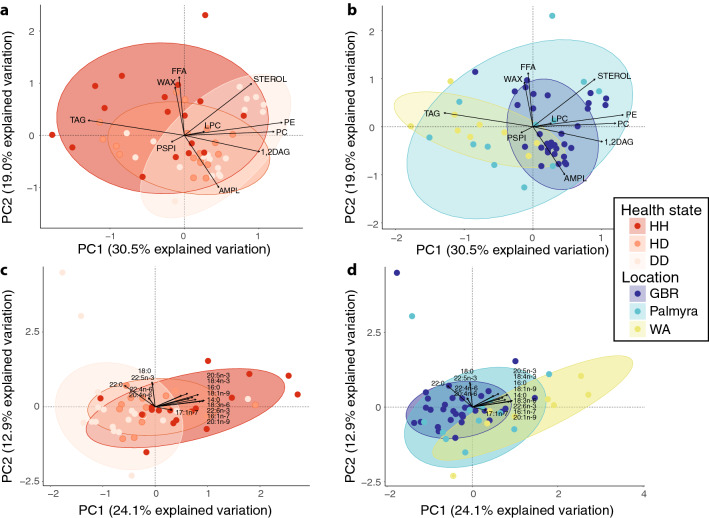
Two-dimensional redundancy analysis fitted with loading vectors for (**A**) lipid class composition visualised by health state; (**B**) lipid class composition visualised by location; (**C**) fatty acid profile visualised by health state; and (**D**) fatty acid profile visualised by location. Ellipses are 90% confidence intervals for each group, and are visualised on the same multivariate space for lipid (**A**,**B**) or fatty acid (**C**,**D**) results. Abbreviations for lipid classes as detailed in methods. This figure was created in R Studio, version 1.2.5033 (https://rstudio.com).

Overall lipid class composition was significantly different between health states (PERMANOVA; df = 2, *F* = 3.39, *p* = 0.017; Fig. 2a; Supplementary Table S3), and regions (df = 2, *F* = 9.10, *p* = 0.0002; Fig. 2b; Supplementary Table S4). There was no significant interaction between the two factors. Pairwise PERMANOVA detected significant differences in lipid class composition between WA and GBR (*p* = 0.003). However, there was also a significant difference in dispersions between all locations (*p* = 0.026), driven by greater dispersion in PA samples, thus these results should be read with caution. The RDA plot showed a tendency for healthy samples to cluster toward the negative side of PC1 and this was correlated with higher concentrations of triacylglycerides (TAG; Fig. 2a). In contrast, diseased samples clustered more toward the positive side of PC1 and were correlated with lipid classes phosphatidylcholine (PC) and phosphatidylethanolamine (PE).

**Figure 3:**
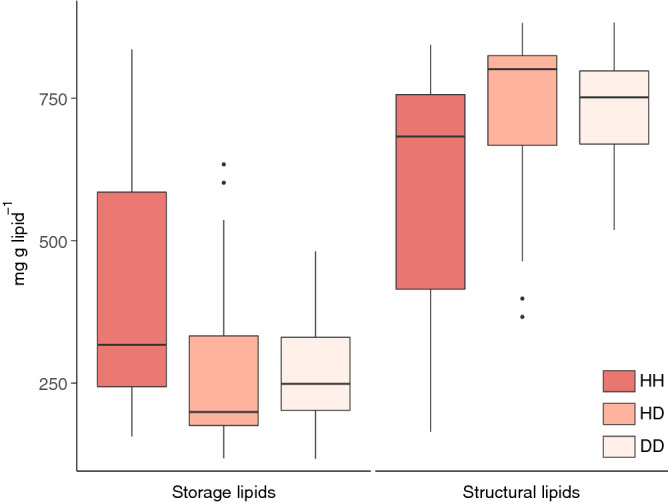
Mean storage and mean structural lipids (mg g lipid^−1^) in healthy and diseased coral colonies. This figure was created in R Studio, version 1.2.5033 (https://rstudio.com).

Storage lipids significantly varied by location (ANOVA; df = 2, F = 10.5, *p* = 0.0002) and health status (df = 2, F = 5.1, *p* = 0.01). However, pairwise comparisons between health states within locations showed significant differences only in PA samples, likely due to high variance in healthy samples. Nonetheless, HH tissue consistently had higher storage lipids than HD and DD tissues, regardless of location (Fig. 3). Conversely, structural lipids showed the reverse trend, with higher concentrations in DD and HD tissues than HH tissues. Overall, structural lipids varied significantly by location (ANOVA, df = 2, F = 10.5, *p* = 0.0002) and health state (df = 2, F = 5.08, *p* = 0.01), though again within location pairwise comparisons of health state were not statistically significant. The lipid classes PC and PE had generally higher concentrations in diseased samples while TAG had generally higher concentrations in healthy samples (Supplementary Table S4).

### Fatty acid composition

Fatty acid composition varied significantly by health state (PERMANOVA; df = 2, F = 5.6, *p* = 0.004; Fig. 2c; Supplementary Table S5) and location (df = 2, F = 7.2, *p* = 0.001; Fig. 2d; Supplementary Table S6), but there was no significant interaction between the two factors. Pairwise comparisons between health states showed a significant difference in fatty acid composition between HH and DD tissues (adjusted *p* = 0.015); while the only significant difference between pairwise location comparisons was between GBR and WA samples (adjusted *p* = 0.006).

Loading vectors of the fatty acids with greatest contribution to between health-state dissimilarity with the ordination plot showed that 18:3n-6 had the highest correlation with the first axis (0.94), while 18:0 had the highest correlation along the second axis (0.83; Fig. 2). Almost all fatty acids showed correlation with the positive side of PC1, which is also where healthy samples clustered. Correlation vectors for two saturated fatty acid (SFA), 18:0 and 22:0, and three polyunsaturated fatty acids (PUFA), 20:4n-6, 22:4n-6, and 22:5n-3 were associated with the negative side of PC1 and in the direction of the diseased sample cluster. All three of these PUFAs had higher mean concentrations in healthy tissues (HH) than diseased tissues (DD; Supplementary Table S5).

### Patterns of bacterial richness and diversity in healthy and diseased corals

**Figure 4:**
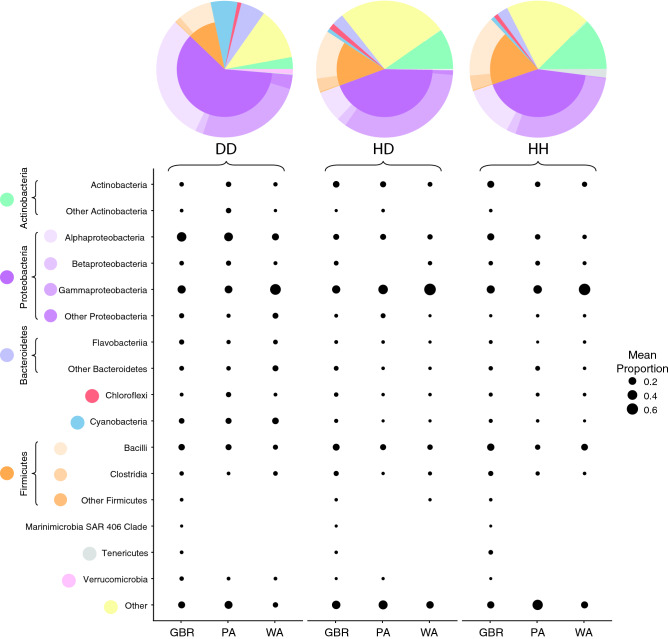
Mean abundance of major taxonomic divisions (phyla and classes) of microbial ASVs recovered from communities within diseased and healthy *A. hyacinthus* colonies from the GBR, PA, and WA. Pie charts represent combined mean abundance across all locations, while bubble charts represent mean abundance within each location. This figure was created in R Studio, version 1.2.5033 (https://rstudio.com).

A total of 401,405 high quality bacterial 16S rRNA gene amplicon reads were generated from coral tissues across all sample types (n = 79), resulting in an average of 4181 reads per sample across 1139 ASVs. Taxonomic divisions across all samples were dominated by Proteobacteria (49% of all reads; mostly Gammaproteobacteria [25%] and Alphaproteobacteria [19%]; Fig. 4), with increased abundances of Alphaproteobacteria, Bacteroidetes, and Cyanobacteria in diseased samples (Fig. 4). There was no significant difference in alpha diversity between sample types (HH, HD, DD; ANOVA, df = 2, *F* = 2.99, *p* = 0.057) or regions (GBR, PA, WA; df = 2, *F* = 2.54, *p* = 0.086). Despite a lack of statistical significance, alpha diversity was consistently higher in diseased tissue (DD; mean ± SE, 2.4 ± 0.1) than healthy tissue on diseased colonies (1.9 ± 0.2) or healthy tissue on healthy colonies (2.1 ± 0.2; Supplementary Fig.S3). Overall microbial community composition differed significantly between health state (PERMANOVA; df = 2, *F* = 2.17, *p* = 0.0001; Supplementary Fig. S4) and region (df = 2, *F* = 1.48, *p* = 0.0059; Supplementary Fig. S4), but the interaction of these factors was not significant. Beta dispersion was not significantly different between health state (df = 2, *F* = 0.83, *p* = 0.44) nor region (df = 2, *F* = 1.6, *p* = 0.21). Pairwise comparisons revealed that microbial communities associated with healthy tissue derived from healthy colonies (HH) were not significantly different from healthy tissue from diseased colonies (HD; df = 1, *F* = 1.01, adjusted *p* = 1.0), but communities associated with both HH and HD were significantly different to diseased tissue (HH:DD: df = 1, *F* = 2.88, *p* = 0.003; HD:DD: df = 1, *F* = 2.48, *p* = 0.003). Pairwise comparisons of regions revealed that microbial communities from the GBR were significantly different from PA (df = 1, *F* = 1.70, *p* = 0.042), but no other pairwise regional microbial community comparisons were significantly different. These significant differences were not easily visualized with the nMDS plots, with 90% confidence intervals of each group overlapping (Supplementary Fig. S4). However, the difference between microbial communities associated with diseased and healthy tissues (i.e. HH:DD and HD:DD) was an order of magnitude greater than the difference between regions. The differences between health states was irrespective of region, indicating that a strong pattern of microbial community structure exists on *A. hyacinthus* corals corresponding to coral health state regardless of geographic location. Interestingly, sequences affiliated with *Halomonas*, which was the 4th most abundant microbial taxon retrieved, was not detected in any diseased tissue samples. In contrast, sequences affiliated to five other microbial taxa (i.e. Cyanobacteria III I, *Arcobacter*, *Rubritalea*, *Rivularia*, and *Labrenzia*) were represented in the top 30 most abundant taxa, but were not present in any healthy tissue (HH or HD) samples.

Because there was no significant difference in overall microbial community structure between healthy tissues from diseased or healthy colonies, these groups were combined for differential abundance analysis to help interpretation of results, as multivariate response linear regression modelling can only be visualised using pairwise comparisons. Regression modelling of balances using health state (HH + HD vs DD) and region explained 10% of variation in microbial community composition, with health state explaining the greatest proportion of the variation (R^2^ = 3.9%). Correlation clustering identified that *Winogradskyella, Ruegeria, Rubritalea,* and 6 sequence variants associated with Rhodobacteraceae were differentially positively abundant in diseased tissue (Supplementary Fig. S5).

## Discussion

Examining WSs from corals distributed across dispersed geographical sites of the Indo-Pacific can help identify patterns that are consistent between lesions and further clarify aspects of the host response to disease. In this study, we provide insight into the energetic reserves and microbial ecology of three tissue types of WSs sampled from plating acroporid colonies from WA, PA, and the GBR. At the gross colony level, all lesions displayed similar disease signs of macroscopic loss of tissue across a broad front resulting in necrotic tissue and exposing irregular bands of white skeleton. Total lipid concentrations displayed a stepwise decrease from HH to HD to DD samples, with healthy samples being characterised by a high proportion of high energy storage lipids. Microbial communities associated with tissues of healthy and diseased areas were distinct when compared between health state and region, supporting previous studies that show corals undergo changes in microbial community composition when affected by WS, and this change is characterised by relative increases in Rhodobacteraceae-affiliated sequences^68,96,97^.

Cnidarian immune responses to disease are, in general, thought to involve the production of antimicrobial peptides and reactive oxygen species to kill bacteria, antioxidants to reduce self-harm, and the accumulation of melanin to prevent the spread of infection^27,98,99^. Each of these processes are energetically expensive and will lead to a reduction of stored reserves. As such, the stepwise decrease in total lipid concentrations from HH to HD to DD tissues, regardless of location, may indicate catabolism of lipid reserves for energy to combat disease progression through such immune responses.

Alternatively, diminished lipid reserves in diseased tissue may also reflect intracolonial transfer of important energy reserves away from lesion sites towards healthy sites. The coral animal is a physiologically integrated collection of individual polyps connected through a shared gastrovascular system, which allows both partitioning and sharing of resources. Lipids have been shown to be transferred from branch bases to tips to contribute to colony growth and calcification^52,100^, and at colony edges, lipids and fatty acids are lowest, suggesting catabolism of these compounds to support tissue synthesis^101^. Lipid translocation in response to WSs would have the combined effect of shutting down the diseased tissue to prevent further resource loss, whilst also fortifying healthy tissues with additional energetic resources^12^. It should also be considered that the capacity to replenish energy reserves via autotrophy or heterotrophy by coral polyps near the lesion interface would be severely reduced, further decreasing lipid concentrations. Neither can we exclude lipid depletion as a result of consumption by opportunistic microbes proliferating in diseased tissues.

A surprising observation in this study was the marked differences in crude lipid quantity between sites, regardless of health state. Of note was that the lowest lipid concentration in WA corals (DD tissue) was comparable to that of healthy tissues (HH) from the GBR. Lipid concentrations are often used as a proxy for coral health. However, if we consider the high concentration of lipids observed in healthy WA samples as a standard, it suggests that no level is sufficiently high to prevent infection and progression of WS disease. In addition, these results support the growing consensus that crude lipid concentrations alone do not provide an accurate proxy for overall coral health. Indeed, lipid concentrations among healthy corals are known to differ in line with a range of geographical and physicochemical factors^102^. Instead, examining patterns among individual lipid classes and fatty acids, as well as ratios between major groups such as storage and structural lipids, can provide more accurate insight to coral health status. Furthermore, future work should incorporate measures of local conditions (e.g. temperature, pH, dissolved oxygen and nutrients, available prey items) as well as other measures of coral health (i.e. Symbiodiniaceae density) to investigate correlations with health metrics.

In the present study, the opacity of the crude lipid results between sites was resolved upon examination of the constituent lipid classes and fatty acids. Trends in lipid classes between HH, HD, and DD tissues were consistent across sites, with storage lipids being lowest in HD and DD tissue, regardless of the total lipid concentration. Storage lipids such as wax esters, triacylglycerols and free fatty acids are high energy compounds that can be rapidly catabolised for ATP production to fuel basal metabolism and energetically-expensive processes during times of stress^103^. For example, colonies of *Porites compressa* and *Montipora capitata* were shown to preferentially catabolise storage lipids when subjected to bleaching conditions^104^. Free fatty acids were particularly depleted in DD samples, indicating that these tissues were consuming lipid reserves even more quickly through a sequential metabolism by the coral host^105^.

The shift toward structural lipids in HD and DD tissues was driven by decreases in triacylglycerol and concurrent increases in phosphatidylcholine and phosphatidylethanolamine. These two phospholipids are major components of biological membranes, and are not thought to be involved in host metabolism^106^. Thus, the predominance of structural lipids in HD and DD corals likely reflects their indispensable roles in cell membrane structure and function. Notably, HD tissues were more similar to DD than to HH tissues in both total lipid concentrations and patterns of storage and structural lipids, demonstrating that WS incites a colony-wide systemic response, with apparently healthy tissues responding to the disease before polyps display visual signs. The mechanisms of this response are as yet unknown, but may include the prophenoloxidase-activating system, as this immune function has also been shown to be suppressed in visually healthy tissues of WS-infected colonies^107^. Resource partitioning may also vary with colony size and distance from the lesion front. HD tissues were collected ~ 10 cm away from the lesion and therefore potentially influenced more than tissues further from the lesion. Future studies should sample over a distance gradient away from the lesion front and combine time-series sampling of apparently healthy colonies prior to and during WS infection to elucidate how lipid levels relate to disease susceptibility.

Consistent with the lipid class results, the fatty acids that were most strongly associated with HH tissues were high energy compounds such as 14:0, 16:0, 18:1n-9, 20:5n-3 and 22:6n-3. In contrast, the fatty acids influencing the separation of the DD tissues were indispensable, membrane-bound compounds such as 18:0, and compounds associated with wound-healing and inflammatory responses, namely 22:5n-3 and 20:4n-6. Arachidonic acid (ARA; 20:4n-6) is the main component of membrane phospholipids and is also one of the primary precursor molecules for the biosynthesis of eicosanoids. Eicosanoids are a complex family of signalling molecules that play a critical role in regulating physiological processes relating to homeostasis and inflammation, such as the production of cytokines and migration of phagocytic cells^108,109^. Docosapentaenoic acid (DPA; 22:5n-3) is an intermediary product between eicosapentaenoic acid (EPA; 20:5n-3) and docosahexaenoic acid (DHA; 22:6n-3), with evidence suggesting it is involved in wound-healing through the migration of cells in vertebrates^110^. However, the role of 22:5n-3 in invertebrate immunity is not yet known. While both 20:4n-6 and 22:5n-3 were shown to influence the separation of the DD tissues from HH tissues using multivariate analyses, the quantitative data also showed they were largely depleted in DD tissues, along with another prominent eicosanoid precursor, eicosapentaenoic acid (20:5n-3), providing evidence that they were oxidised to eicosanoids in diseased sites and catabolised to fuel the immune response. Furthermore, the maintenance of high levels of PC and PE in diseased tissue coupled with low levels of 20:4n-6 and 20:5n-3 suggest that the depletion of 20:4n-6 and 20:5n-3 is caused by breakdown and usage of these fatty acids rather than impaired membrane structure. However, their catabolism for other metabolic processes cannot be completely discounted at this point in time, with further concomitant investigations into circulating eicosanoid levels required to shed light on this topic.

Previous histological analyses of samples from the GBR^68^ reported extensive necrosis and tissue degeneration in disease lesions, and comparison of these samples with additional samples from WA and PA confirmed these patterns. For example, only some WS lesion tissues displayed the presence of ciliate and fungal cells and few signs of tissue necrosis, swelling, abnormalities or microbial colonization were apparent in the healthy tissues preceding the lesion front (~ 1 mm in front of lesion). While we cannot categorically rule out the role of microbial communities or viruses in disease onset and/or progression based on histological evidence alone, the low densities of ciliates and fungi across the disease tissue samples from the three sites suggests that they are unlikely to be causative agents of WS at these sites, and rather are secondary invaders following initial infection. While previous work has implicated ciliate histophagy as a form of secondary pathogenesis following bacterial challenge, which is required to produce the tissue loss patterns characteristic of WS^21^, we would expect to identify ciliates in 100% of samples if this were the case. However, histological and FISH approaches may not be adequate to detect all microbial entities present, and other diagnostic techniques would more fully resolve the microbial community dynamics of WS. Helminths were present in even lower abundances but were also present in healthy tissue, and are therefore also unlikely to be epidemiologically relevant, but rather opportunistic or parasitic settlers within compromised coral tissue. Aligning with previous work on GBR samples^68^, FISH analysis confirmed the presence of bacteria within all WA disease lesions, while no bacteria were visualised in healthy tissue (with the exception of coral-associated microbial aggregates; CAMAs^95^), even within 1 mm of the lesion boundary. Bacterial signal was much higher in the compromised, necrosing tissue behind the lesion front, supporting the hypothesis that bacteria are secondary, opportunistic colonisers rather than drivers of disease. *Vibrio* bacteria were detected in a small proportion of lesions but were visualised in much lower abundance than total bacteria, and thus are also unlikely to drive WS causation. Our results contrast with previous studies that identify *Vibrio* bacteria^111^, ciliates^20,21^, viruses^22^, and helminths^23^ as potential causative agents, highlighting that the cause of WSs are likely multifaceted and thus adding to the evidence that WSs encompasses various distinct aetiologies.

Though there was no single dominant bacterial taxon associated with disease lesions from the disparate sampling sites to provide any indication of a specific bacterial agent linked to WS causation, there were differences in microbial community composition between healthy and diseased tissues. Healthy tissue from healthy colonies (HH) and healthy tissue from diseased colonies (HD) showed no consistent differences in microbial community composition. However, both these tissue types were distinct from diseased tissue (DD), which had consistently higher within-sample bacterial community diversity than healthy tissues. While the differences in within-sample diversity observed here were not statistically distinguishable, fluctuations in diversity have been proposed to underlie biological and ecological stability (i.e. the Anna Karenina principle)^112^. Hence, a decrease in microbiome diversity can be linked with negative impacts on the host^107,112,113^. However, an increase in diversity also represents potential opportunistic colonization and dysbiosis^64^, especially following host tissue mortality as seen in WSs. The sequences recovered from the disease lesions across the three sampling sites indicate this is the case with compromised and necrosing diseased tissues likely supporting a diverse and variable opportunistic bacterial community.

Diseased tissue was characterised by relative increases in Cyanobacteria and Bacteroidetes, and was dominated by Proteobacteria with increased abundance of Alphaproteobacteria (particularly Rhodobacteraceae) compared to healthy tissue. These observations were consistent across the three sampling sites and align with previous findings that a distinct microbiome occurs at the lesion front, characterized by a positive differential abundance of Rhodobacteraceae-affiliated sequences^68,96,97,114^. The family Rhodobacteraceae is emerging as a potential indicator of compromised coral health, with reports of elevated levels in WSs^68,96,97,114^ as well as several other coral diseases^115–118^. In this study, the significant increase in Rhodobacteraceae in diseased tissue across locations supports previous work that proposes this group to be associated with compromised health. Rhodobacteraceae-affiliated sequences represented nearly 20% of all disease sample sequences, while representing 12% of HH colony sequences and only 3% of HD colony sequences. However, the decrease in Rhodobacteraceae between HH colonies and HD colonies suggests the relationship of this family of bacteria to disease is not straightforward. Indeed, this group has been frequently identified as core members of the coral microbiome^119–121^, but is also implicated in a variety of stress responses^96,122–124^. It is likely, therefore, that this group comprises a combination of commensal, as well as opportunistic and potentially pathogenic members. It is possible that the observed decrease in Rhodobacteraceae from HH to HD tissue represents a loss of commensal community members, with the subsequent increase in DD tissue as opportunistic colonization of compromised tissue occurs. Similarly, Bacteroidetes have been implicated in diseased sponges and corals^125,126^ including white plague infected colonies in Brazil^127–129^, but also have been associated with secondary colonisation following tissue mortality^125^. Bacteroidetes preferentially consume high molecular weight organic matter^130^, and so it is likely that necrotic tissues provide a varied source of nutrients and may drive the increase in Bacteroidetes abundance in diseased tissue^126^. In this study, however, a large proportion of Bacteroidetes ASVs failed to classify below Class level and thus no specific taxa can be pinpointed, and thus it remains unclear if these are associated with diseased tissue or skeletal overgrowth.

Interestingly, a complete loss of sequences associated to the genus *Halomonas* (Gammaproteobacteria) was observed between healthy to diseased tissue. *Halomonas* spp. have been implicated in the metabolism of DMSP and its breakdown product acrylic acid, which may generate antimicrobial compounds such as TDA, and thus *Halomonas* has been proposed as a potential member of a probiotic consortia for microbiome engineering^131–133^. Future investigations into these taxa, including how their abundance changes over the course of disease onset and their role in metabolism, may be of particular interest especially in the context of reef restoration initiatives^131^.

In conclusion, the underlying causation of white syndromes of acroporid corals of the Indo-Pacific remains elusive. However, here we show that the coral energetic response is similar across locations and is characterized by a stepwise loss of high energy storage lipids from healthy to diseased tissue. This agrees with previous studies which have profiled coral responses to other kinds of stress (such as bleaching and ocean acidification), whereby corals utilize their energy reserves to launch an immune response. Visually healthy tissue from diseased colonies (HD) had lipid patterns more similar to diseased tissue (DD) than to healthy tissue from visually unaffected colonies (HH). The similarities in lipid patterns between HD and DD tissues are signatures of a systemic response, suggesting that WS depletes resources across an entire colony through polyp connective tissue, with lipids likely being catabolised to launch an immune response. Importantly, we have demonstrated that examining the qualitative aspects of lipids in diseased corals is required to gain comprehensive insight into their health. Several studies have relied on total lipid quantification alone as a measure of health^49,103,134,135^. Here we demonstrate that the total lipid in diseased corals from WA is akin to that of healthy corals from the GBR, and thus no minimum lipid level can be used to characterize coral health across locations. Therefore, while high lipid stores have been shown to mitigate adverse effects of warming and acidification, the same does not appear to be true for disease. Further studies are needed to define local standards of lipid biomarkers for coral health, specifically how these relate to autotrophic and heterotrophic energy acquisition. Investigation into fatty acids also revealed low levels of ARA and EPA in diseased tissue, suggesting oxidation of these compounds to eicosanoids may be occurring to combat infection. The role of specific fatty acids in coral immune response is poorly understood and merits further research. We detected increased abundance of Rhodobacteraceae-associated sequences, which aligns with previous studies identifying this group as colonising compromised coral tissues, though its role in pathogenesis is unknown. The loss in relative abundance of *Halomonas* from healthy tissues provides further support for the role of these microbes in coral health. To further elucidate the interaction of environment, host energetics and microbes in WS onset and progression, high resolution time-series sampling may aid in identifying the contributing factors that ultimately manifest as lesions on acroporid coral colonies across Indo-Pacific reefs.

## Supplementary information

Supplementary information.

## Data Availability

The datasets supporting this article are available through GenBank Sequence Read Archive under BioProject number PRJNA524926.
