# Supplementary material for: Energy depletion and opportunistic microbial colonisation in white syndrome lesions from corals across the Indo-Pacific
Source: Sci Rep. 2020 Nov 17;10:19990. doi: 10.1038/s41598-020-76792-x (PMC7672225; doi:10.1038/s41598-020-76792-x)
Supplement: Supplementary file 1 — Supplementary information. [file 41598_2020_76792_MOESM1_ESM.pdf]

ENERGY DEPLETION AND OPPORTUNISTIC MICROBIAL COLONISATION IN WHITE SYNDROME LESIONS FROM CORALS ACROSS THE INDO-PACIFIC

Hillary A. Smith, Jessica A. Conlan, F. Joseph Pollock, Naohisa Wada, Amanda Shore, Julia Yun-Hsuan Hung, Greta S. Aeby, Bette L. Willis, David S. Francis, David G. Bourne

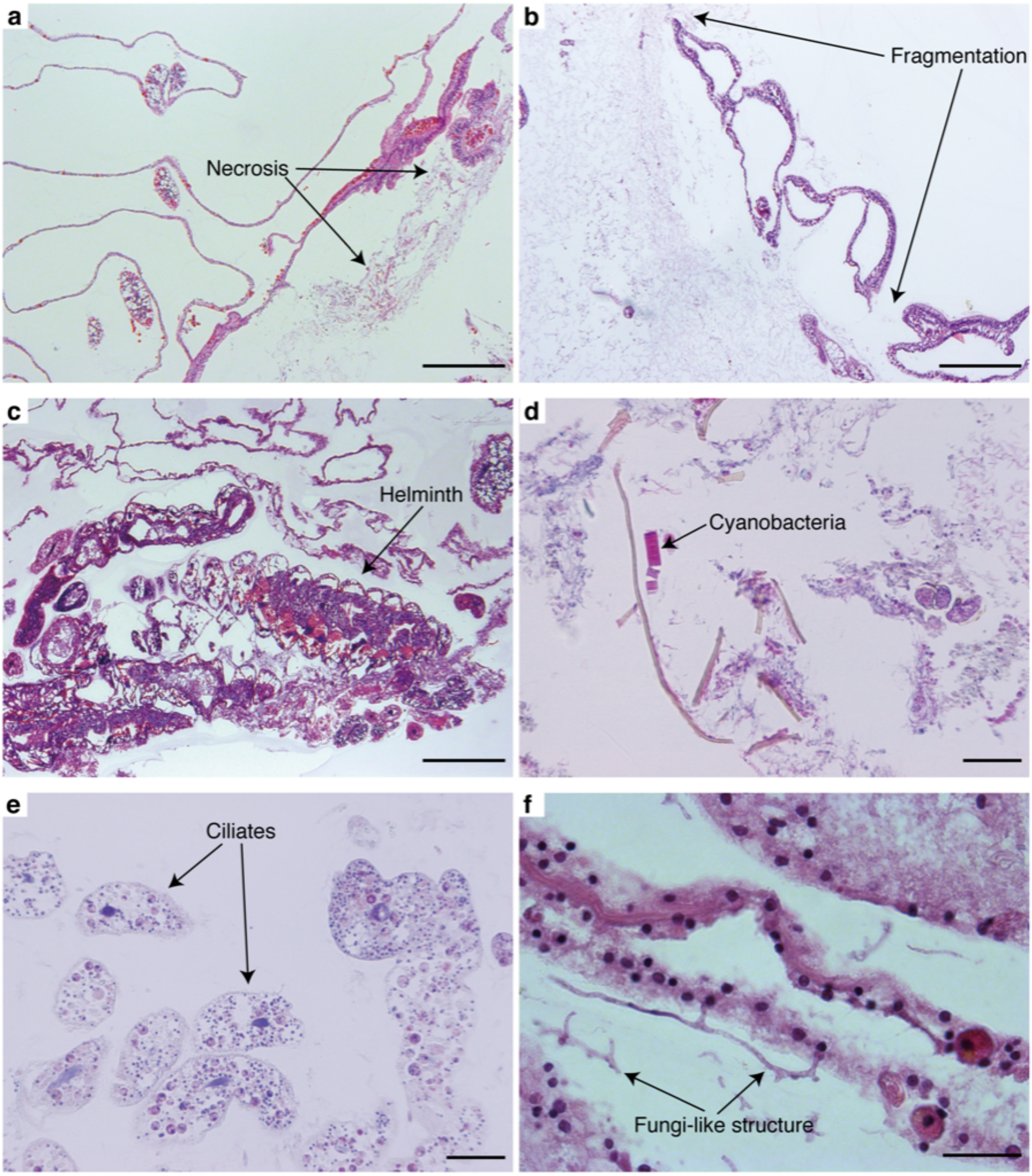

Supplementary Figure S1. Histological images of a) tissue necrosis (scale bar = 200 µm); b) fragmentation (scale bar = 200 µm); c) helminths (scale bar = 200 µm); d) cyanobacteria (scale bar = 50 µm); e) ciliates (scale bar = 50 µm); and f) fungi-like structures (scale bar = 20 µm) detected in colonies of WS-diseased *Acropora hyacinthus.*

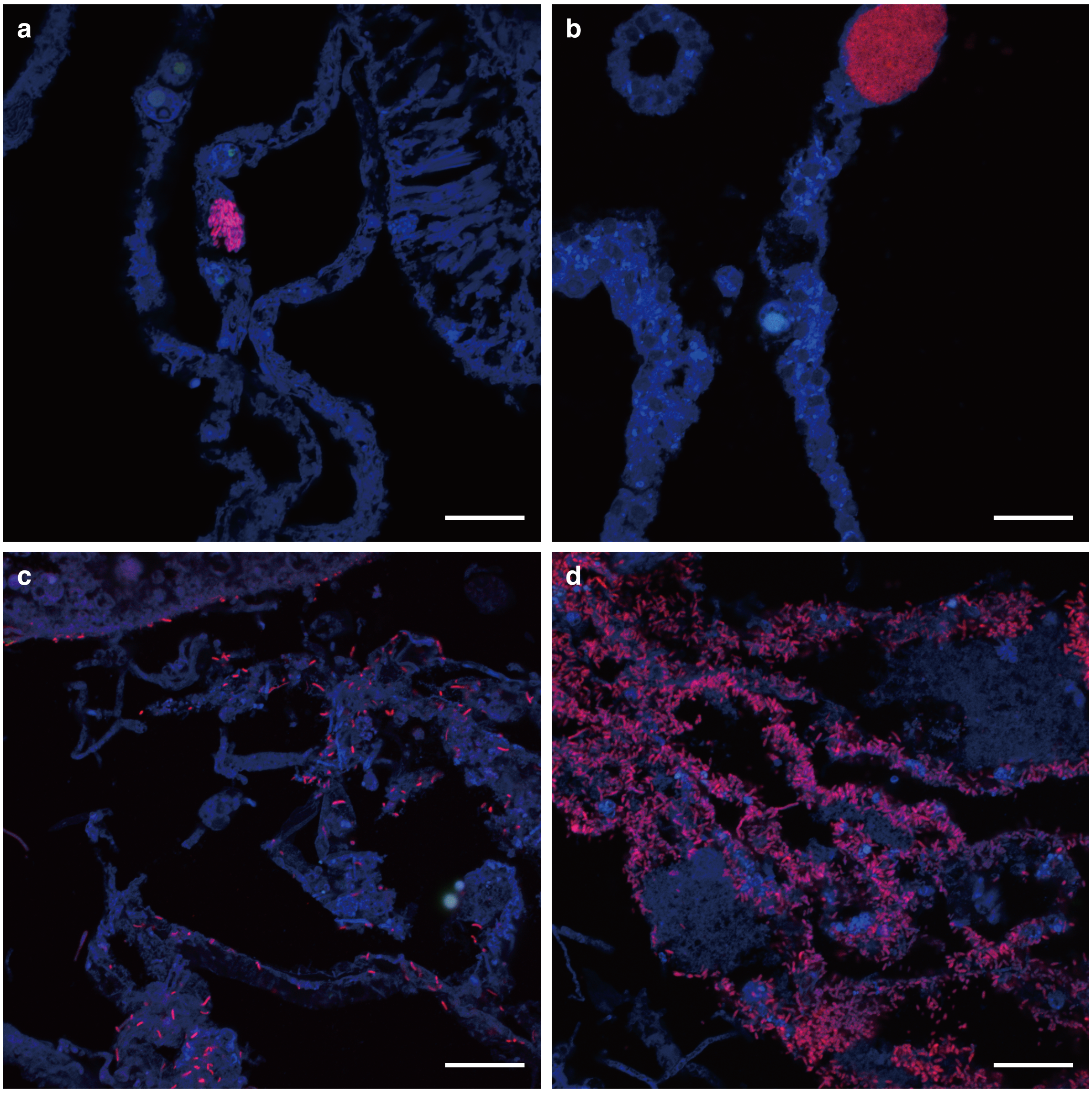

Supplementary Figure S2. Fluorescence in situ hybridisation micrographs of *Acropora hyacinthus* from a) HH tissue; b) HD tissue, D1 region; c) the lesion front, D2 region; and d) DD tissue, D3 region. Scale bars = 20 µm).

Supplementary Table S1. Comparison of proximate composition of samples types within locations. Values are presented as means ± SEM. Values in the same row and group (i.e. location) that do not share the same superscripts are significantly different (p < 0.05).

	WA			PA			GBR		
(mg g sample^{-1})	**DD**	**HD**	**HH**	**DD**	**HD**	**HH**	**DD**	**HD**	**HH**
**Ash**	958 ± 4.67^{a}	942 ± 5.48^{b}	910 ± 14.6^{c}	964 ± 1.61^{a}	945 ± 9.71^{ab}	934 ± 11^{b}	964 ± 2.31^{a}	952 ± 5.72^{b}	949 ± 2.4^{b}
**AFDW**	158 ± 24.9^{b}	285 ± 34.3^{a}	276 ± 55.3^{ab}	114 ± 31^{a}	129 ± 29.6^{a}	223 ± 54.3^{a}	76.9 ± 11.5^{b}	113 ± 15.2^{a}	134 ± 10.7^{a}
**Lipid** *(mg g AFDW^{-1})*	6.89 ± 1.93^{b}	17.2 ± 3.5^{a}	23.6 ± 2.74^{a}	4.15 ± 1.17^{b}	7.78 ± 3.18^{ab}	15.3 ± 4.84^{a}	2.76 ± 0.44^{c}	5.07 ± 0.74^{b}	6.79 ± 0.54^{a}

Supplementary Table S2. Comparison of proximate composition of sample types between locations. Values are presented as means ± SEM. Values in the same row and group (i.e. sample type) that do not share the same superscripts are significantly different (p < 0.05).

	DD			HD			HH		
(mg g sample^{-1})	**GBR**	**PA**	**WA**	**GBR**	**PA**	**WA**	**GBR**	**PA**	**WA**
**Ash**	964 ± 2.31^{a}	964 ± 1.61^{ab}	958 ± 4.67^{b}	952 ± 5.72^{a}	945 ± 9.71^{a}	942 ± 5.48^{a}	949 ± 2.4^{a}	934 ± 11^{b}	910 ± 14.6^{b}
**AFDW**	76.9 ± 11.5^{b}	114 ± 31^{ab}	158 ± 24.9^{a}	113 ± 15.2^{b}	129 ± 29.6^{b}	285 ± 34.3^{a}	134 ± 10.7^{b}	223 ± 54.3^{ab}	276 ± 55.3^{a}
**Lipid** *(mg g AFDW^{-1})*	2.76 ± 0.44^{b}	4.15 ± 1.17^{ab}	6.89 ± 1.93^{a}	5.07 ± 0.74^{b}	7.78 ± 3.18^{b}	17.2 ± 3.5^{a}	6.79 ± 0.54^{b}	15.3 ± 4.84^{ab}	23.6 ± 2.74^{a}

Supplementary Table S3. Comparison of lipid class composition (mg g lipid^{-1}) between sample types within locations. Values are presented as means ± SEM. Values in the same row and group (i.e. location) that do not share the same superscripts are significantly different ($p < 0.05$).

	WA			Palmyra			GBR		
**Class**	**DD**	**HD**	**HH**	**DD**	**HD**	**HH**	**DD**	**HD**	**HH**
**WAX**	83.6 ± 13.7^{a}	86.9 ± 15.9^{a}	114 ± 75.5^{a}	82 ± 18.2^{a}	76.4 ± 13^{a}	62.5 ± 21.2^{a}	82.2 ± 7.04^{b}	76.5 ± 11^{b}	139 ± 20.9^{a}
**TAG**	344 ± 131^{a}	492 ± 77.7^{a}	462 ± 169^{a}	97.7 ± 42.5^{a}	110 ± 44.9^{a}	325 ± 150^{a}	56.1 ± 6.5^{a}	73.9 ± 21.8^{a}	117 ± 32.2^{a}
**FFA**	24 ± 3.23^{a}	30.8 ± 4.36^{a}	17.9 ± 6.13^{a}	26.3 ± 7.32^{a}	26.6 ± 7.29^{a}	62.3 ± 32^{a}	20.4 ± 3.13^{b}	25.1 ± 4.47^{b}	38.4 ± 5.05^{a}
**1,2DAG**	17.5 ± 17.5^{a}	0 ± 0^{a}	9.34 ± 9.34^{a}	11.5 ± 11.5^{a}	35.4 ± 20.4^{a}	7.93 ± 7.93^{a}	105 ± 6.91^{a}	45.4 ± 10.1^{b}	32.3 ± 11.6^{b}
**STEROL**	43.6 ± 6.14^{a}	33.2 ± 3.44^{a}	44.7 ± 9.95^{a}	65.9 ± 17^{a}	61.3 ± 12.9^{a}	71.5 ± 38.3^{a}	64.2 ± 5.49^{a}	66.4 ± 3.42^{a}	68.2 ± 4^{a}
**AMPL**	353 ± 93.8^{a}	250 ± 66.5^{a}	229 ± 108^{a}	446 ± 103^{a}	469 ± 33.9^{a}	370 ± 132^{a}	414 ± 35.8^{a}	461 ± 33.9^{a}	380 ± 37.6^{a}
**PE**	46.7 ± 16^{a}	42.6 ± 7.48^{a}	60.3 ± 32.2^{a}	104 ± 29.7^{a}	76.7 ± 15.2^{a}	60.9 ± 16.9^{a}	113 ± 6.64^{a}	90.7 ± 5.49^{b}	74.1 ± 6.05^{b}
**PSPI**	10.4 ± 10.4^{a}	11.8 ± 11.8^{a}	5.67 ± 5.67^{a}	0 ± 0^{a}	39.1 ± 27.2^{a}	4.29 ± 4.29^{a}	0 ± 0^{a}	27.7 ± 16.1^{a}	18.7 ± 9.96^{a}
**PC**	76.7 ± 11.4^{a}	53.3 ± 9.46^{a}	56.9 ± 16.9^{a}	127 ± 35.8^{a}	103 ± 21.9^{ab}	35.3 ± 15.3^{b}	135 ± 6.83^{a}	133 ± 6.45^{a}	125 ± 8.68^{a}
**LPC**	0 ± 0	0 ± 0	0 ± 0	39.2 ± 28.9^{a}	3.18 ± 3.18^{a}	0 ± 0^{a}	10.8 ± 7.37^{a}	0 ± 0^{a}	7.03 ± 7.03^{a}
**∑STORAGE**	469 ± 115^{a}	609 ± 84.1^{a}	604 ± 161^{a}	218 ± 45.6^{a}	248 ± 41.6^{a}	458 ± 147^{a}	263 ± 21.2^{ab}	221 ± 35.2^{b}	327 ± 38^{a}
**∑STRUCTURAL**	531 ± 115^{a}	391 ± 84.1^{a}	396 ± 161^{a}	782 ± 45.6^{a}	752 ± 41.6^{a}	542 ± 147^{a}	737 ± 21.2^{ab}	779 ± 35.2^{a}	673 ± 38^{b}
**RATIO**	1.38 ± 0.79^{a}	2.01 ± 0.75^{a}	2.55 ± 1.36^{a}	0.29 ± 0.08^{a}	0.34 ± 0.08^{a}	1.41 ± 0.72^{a}	0.37 ± 0.04^{ab}	0.32 ± 0.09^{b}	0.54 ± 0.11^{a}

Supplementary Table S4. Comparison of lipid class composition (mg g lipid^{-1}) of sample types between locations. Values are presented as means ± SEM. Values in the same row and group (i.e. sample type) that do not share the same superscripts are significantly different ($p < 0.05$).

	DD			HD			HH		
**Class**	**GBR**	**Palmyra**	**WA**	**GBR**	**Palmyra**	**WA**	**GBR**	**Palmyra**	**WA**
**WAX**	82.2 ± 7.04^{a}	82 ± 18.2^{a}	83.6 ± 13.7^{a}	76.5 ± 11^{a}	76.4 ± 13^{a}	86.9 ± 15.9^{a}	139 ± 20.9^{a}	62.5 ± 21.2^{b}	114 ± 75.5^{ab}
**TAG**	56.1 ± 6.5^{b}	97.7 ± 42.5^{b}	344 ± 131^{a}	73.9 ± 21.8^{b}	110 ± 44.9^{b}	492 ± 77.7^{a}	117 ± 32.2^{b}	325 ± 150^{ab}	462 ± 169^{a}
**FFA**	20.4 ± 3.13^{a}	26.3 ± 7.32^{a}	24 ± 3.23^{a}	25.1 ± 4.47^{a}	26.6 ± 7.29^{a}	30.8 ± 4.36^{a}	38.4 ± 5.05^{a}	62.3 ± 32^{a}	17.9 ± 6.13^{b}
**1,2DAG**	105 ± 6.91^{a}	11.5 ± 11.5^{b}	17.5 ± 17.5^{b}	45.4 ± 10.1^{a}	35.4 ± 20.4^{ab}	0 ± 0^{b}	32.3 ± 11.6^{a}	7.93 ± 7.93^{a}	9.34 ± 9.34^{a}
**STEROL**	64.2 ± 5.49^{a}	65.9 ± 17^{a}	43.6 ± 6.14^{a}	66.4 ± 3.42^{a}	61.3 ± 12.9^{a}	33.2 ± 3.44^{b}	68.2 ± 4^{a}	71.5 ± 38.3^{a}	44.7 ± 9.95^{a}
**AMPL**	414 ± 35.8^{a}	446 ± 103^{a}	353 ± 93.8^{a}	461 ± 33.9^{a}	469 ± 33.9^{a}	250 ± 66.5^{b}	380 ± 37.6^{a}	370 ± 132^{a}	229 ± 108^{a}
**PE**	113 ± 6.64^{a}	104 ± 29.7^{ab}	46.7 ± 16^{b}	90.7 ± 5.49^{a}	76.7 ± 15.2^{a}	42.6 ± 7.48^{b}	74.1 ± 6.05^{a}	60.9 ± 16.9^{a}	60.3 ± 32.2^{a}
**PSPI**	0 ± 0^{a}	0 ± 0^{a}	10.4 ± 10.4^{a}	27.7 ± 16.1^{a}	39.1 ± 27.2^{a}	11.8 ± 11.8^{a}	18.7 ± 9.96^{a}	4.29 ± 4.29^{a}	5.67 ± 5.67^{a}
**PC**	135 ± 6.83^{a}	127 ± 35.8^{ab}	76.7 ± 11.4^{b}	133 ± 6.45^{a}	103 ± 21.9^{ab}	53.3 ± 9.46^{b}	125 ± 8.68^{a}	35.3 ± 15.3^{b}	56.9 ± 16.9^{b}
**LPC**	10.8 ± 7.37^{a}	39.2 ± 28.9^{a}	0 ± 0^{a}	0 ± 0^{a}	3.18 ± 3.18^{a}	0 ± 0^{a}	7.03 ± 7.03^{a}	0 ± 0^{a}	0 ± 0^{a}
**∑STORAGE**	263 ± 21.2^{b}	218 ± 45.6^{b}	469 ± 115^{a}	221 ± 35.2^{b}	248 ± 41.6^{b}	609 ± 84.1^{a}	327 ± 38^{a}	458 ± 147^{a}	604 ± 161^{a}
**∑STRUCTURAL**	737 ± 21.2^{a}	782 ± 45.6^{a}	531 ± 115^{b}	779 ± 35.2^{a}	752 ± 41.6^{a}	391 ± 84.1^{b}	673 ± 38^{a}	542 ± 147^{a}	396 ± 161^{a}
**RATIO**	0.37 ± 0.04^{b}	0.29 ± 0.08^{b}	1.38 ± 0.79^{a}	0.32 ± 0.09^{b}	0.34 ± 0.08^{b}	2.01 ± 0.75^{a}	0.54 ± 0.11^{a}	1.41 ± 0.72^{a}	2.55 ± 1.36^{a}

Supplementary Table S5. Comparison of fatty acid composition (mg g lipid^{-1}) between sample types within locations. Values are presented as means ± SEM. Values in the same row and group (i.e. location) that do not share the same superscripts are significantly different ($p < 0.05$).

	WA			Palmyra			GBR		
**Fatty acid**	**DD**	**HD**	**HH**	**DD**	**HD**	**HH**	**DD**	**HD**	**HH**
**6:0**	0.15 ± 0.07^{a}	0.06 ± 0.04^{a}	0.02 ± 0.02^{a}	0.13 ± 0.13^{a}	0.16 ± 0.1^{a}	0.09 ± 0.05^{a}	0.39 ± 0.12^{a}	0.14 ± 0.05^{a}	0.13 ± 0.03^{a}
**8:0**	0.01 ± 0.01^{a}	0.03 ± 0.02^{a}	0 ± 0^{a}						
**10:0**	0.07 ± 0.03^{b}	0.27 ± 0.12^{a}	0.14 ± 0.04^{ab}	0 ± 0^{a}	0.02 ± 0.02^{a}	0.03 ± 0.02^{a}	0.01 ± 0.01^{b}	0.06 ± 0.03^{b}	2.14 ± 0.94^{a}
**11:0**	0.01 ± 0.01^{a}	0 ± 0^{a}	0.04 ± 0.02^{a}	0 ± 0^{a}	0.02 ± 0.02^{a}	0.01 ± 0.01^{a}	0 ± 0^{a}	0 ± 0^{a}	0.02 ± 0.02^{a}
**12:0**	0.56 ± 0.16^{a}	0.55 ± 0.03^{a}	0.77 ± 0.16^{a}	0.34 ± 0.2^{a}	0.48 ± 0.05^{a}	0.32 ± 0.04^{a}	1.05 ± 0.26^{a}	0.88 ± 0.12^{a}	1.04 ± 0.13^{a}
**13:0**	0.21 ± 0.05^{a}	0.16 ± 0.03^{a}	0.16 ± 0.09^{a}	0.13 ± 0.07^{a}	0.32 ± 0.07^{a}	0.26 ± 0.11^{a}	0.08 ± 0.04^{b}	0.25 ± 0.03^{a}	0.26 ± 0.07^{a}
**14:0**	14 ± 3.95^{a}	19.9 ± 2.18^{a}	16.6 ± 4.28^{a}	10.1 ± 3.86^{a}	14.7 ± 2.39^{a}	18.6 ± 5.14^{a}	7.39 ± 0.72^{b}	8.88 ± 0.87^{ab}	12.8 ± 2.15^{a}
**15:0**	0.34 ± 0.1^{a}	0.22 ± 0.01^{a}	0.15 ± 0.03^{a}	0.25 ± 0.11^{a}	0.31 ± 0.05^{a}	0.35 ± 0.17^{a}	0.64 ± 0.09^{a}	0.4 ± 0.05^{b}	0.39 ± 0.04^{b}
**16:0**	160 ± 53.4^{a}	221 ± 45^{a}	201 ± 53^{a}	97.7 ± 26.3^{a}	114 ± 19.9^{a}	165 ± 44.1^{a}	76.2 ± 7.47^{b}	82.1 ± 9.97^{b}	121 ± 15.6^{a}
**17:0**	0.28 ± 0.14^{a}	0.52 ± 0.14^{a}	0.46 ± 0.16^{a}	1.83 ± 1.03^{a}	0.31 ± 0.09^{a}	0.44 ± 0.23^{a}	1.68 ± 0.31^{a}	0.56 ± 0.12^{b}	0.44 ± 0.13^{b}
**18:0**	17.7 ± 3.61^{a}	23.6 ± 3.88^{a}	22.3 ± 3.31^{a}	32.3 ± 9.23^{a}	25.7 ± 3.42^{a}	30.3 ± 5.89^{a}	25.3 ± 3.71^{a}	28.2 ± 1.5^{a}	27.9 ± 1.23^{a}
**20:0**	3.12 ± 0.68^{a}	3.7 ± 0.22^{a}	3.2 ± 0.42^{a}	3.62 ± 0.88^{a}	3.54 ± 0.48^{a}	4.36 ± 0.94^{a}	3.91 ± 0.33^{a}	3.66 ± 0.2^{a}	3.82 ± 0.19^{a}
**21:0**	0.48 ± 0.22^{a}	0.22 ± 0.06^{a}	0.18 ± 0.05^{a}	1.16 ± 0.63^{a}	0.37 ± 0.04^{a}	0.27 ± 0.06^{a}	0.99 ± 0.17^{a}	0.54 ± 0.06^{b}	0.39 ± 0.05^{b}
**22:0**	1.45 ± 0.33^{a}	1.25 ± 0.46^{a}	1.16 ± 0.34^{a}	7.39 ± 4^{a}	1.95 ± 0.15^{a}	1.38 ± 0.52^{a}	5.69 ± 1.11^{a}	2.8 ± 0.34^{b}	2.34 ± 0.43^{b}
**24:0**	0.64 ± 0.13^{a}	0.43 ± 0.11^{a}	0.37 ± 0.11^{a}	3.26 ± 1.66^{a}	0.72 ± 0.12^{a}	0.56 ± 0.26^{a}	2.81 ± 0.66^{a}	1.25 ± 0.2^{b}	0.88 ± 0.16^{b}
**14:1n-5**	0.4 ± 0.19^{a}	0.66 ± 0.29^{a}	0.41 ± 0.36^{a}	0.4 ± 0.27^{a}	0.83 ± 0.24^{a}	0.26 ± 0.09^{a}	1.64 ± 0.6^{a}	1.77 ± 0.34^{a}	1.43 ± 0.33^{a}
**15:1n-5**	0.48 ± 0.12^{a}	0.68 ± 0.14^{a}	0.67 ± 0.1^{a}	0.18 ± 0.11^{a}	0.56 ± 0.07^{a}	0.32 ± 0.15^{a}	1 ± 0.35^{a}	1.08 ± 0.23^{a}	1.05 ± 0.25^{a}
**16:1n-7**	14.6 ± 6.47^{a}	19.3 ± 6.86^{a}	16.6 ± 5.8^{a}	2.82 ± 1.08^{a}	4.65 ± 0.61^{a}	6.29 ± 1.56^{a}	3.05 ± 0.25^{a}	3.3 ± 0.28^{a}	5.78 ± 1.32^{a}
**17:1n-7**	23 ± 6.88^{a}	10.9 ± 10.9^{a}	3.99 ± 3.99^{a}	6.88 ± 5.2^{a}	17.6 ± 4.67^{a}	19.4 ± 8.83^{a}	8.62 ± 0.94^{a}	11.6 ± 1.9^{a}	17.9 ± 5.19^{a}
**18:1n-7**	1.25 ± 0.3^{a}	1.09 ± 0.31^{a}	0.98 ± 0.29^{a}	0.32 ± 0.11^{b}	0.46 ± 0.06^{b}	2.41 ± 1.38^{a}	1.4 ± 0.27^{a}	2.07 ± 1.48^{b}	0.61 ± 0.08^{b}
**18:1n-7t**	0.03 ± 0.02^{b}	0.04 ± 0.04^{b}	0.5 ± 0.2^{a}	0 ± 0^{a}	0.07 ± 0.04^{a}	0.19 ± 0.12^{a}	0.01 ± 0.01^{a}	0.05 ± 0.05^{a}	0.1 ± 0.06^{a}
**18:1n-9**	23.6 ± 9.22^{a}	31 ± 10.5^{a}	31.3 ± 10.2^{a}	6.82 ± 1.31^{b}	6.89 ± 1.13^{b}	13.8 ± 2.38^{a}	7.71 ± 0.55^{b}	13 ± 5.89^{b}	14.4 ± 2.71^{a}
**18:1n-9t**	0.01 ± 0.01^{b}	0.24 ± 0.11^{a}	0.51 ± 0.24^{a}	0 ± 0^{b}	0.11 ± 0.09^{b}	1.43 ± 1.16^{a}	0.11 ± 0.11^{a}	4.57 ± 2.98^{a}	0.22 ± 0.13^{a}

**20:1n-9**	7.81 ± 2.51^{a}	5.45 ± 3.63^{a}	9.8 ± 2.13^{a}	2.12 ± 0.43^{a}	3.05 ± 0.61^{a}	4.48 ± 1.45^{a}	2.93 ± 0.27^{b}	3.82 ± 0.4^{b}	5.57 ± 0.59^{a}
**22:1n-9**	0.52 ± 0.11^{a}	0.38 ± 0.03^{a}	0.72 ± 0.22^{a}	4.37 ± 1.99^{a}	1.53 ± 0.33^{ab}	1.43 ± 0.66^{b}	2.56 ± 0.62^{a}	1.2 ± 0.28^{b}	0.96 ± 0.34^{b}
**24:1n-9**	0.18 ± 0.12^{a}	0.07 ± 0.02^{a}	0.11 ± 0.05^{a}	0 ± 0^{a}	0.08 ± 0.06^{a}	0.05 ± 0.04^{a}	0.04 ± 0.03^{a}	0.01 ± 0.01^{a}	0.07 ± 0.03^{a}
**20:1n-11**	0.46 ± 0.25^{a}	0.48 ± 0.27^{a}	0.64 ± 0.22^{a}						
**22:1n-11**	0.23 ± 0.07^{a}	0.15 ± 0.04^{a}	0.15 ± 0.03^{a}	0.13 ± 0.09^{a}	0.12 ± 0.06^{a}	0.09 ± 0.03^{a}	0.28 ± 0.1^{a}	0.24 ± 0.14^{a}	0.13 ± 0.02^{a}
**18:3n-3**	0.21 ± 0.05^{a}	0.15 ± 0.04^{a}	0.13 ± 0.02^{a}	0.02 ± 0.02^{a}	0.02 ± 0.02^{a}	0.06 ± 0.04^{a}	0.31 ± 0.12^{a}	0.16 ± 0.09^{a}	0.1 ± 0.03^{a}
**18:4n-3**	5.14 ± 1.83^{a}	9.04 ± 0.84^{a}	7.84 ± 1.34^{a}	4.71 ± 0.37^{a}	7.99 ± 1.67^{a}	6.14 ± 1.27^{a}	3.8 ± 0.46^{b}	6.43 ± 0.7^{a}	6.66 ± 0.61^{a}
**20:4n-3**	0.44 ± 0.17^{a}	0.7 ± 0.16^{a}	0.46 ± 0.23^{a}	0 ± 0^{a}	0.12 ± 0.12^{a}	0.43 ± 0.25^{a}	0.08 ± 0.05^{b}	0.22 ± 0.08^{ab}	0.42 ± 0.1^{a}
**20:5n-3**	15.8 ± 3.82^{a}	24.2 ± 2.73^{a}	23.8 ± 0.68^{a}	18.6 ± 3.17^{a}	29 ± 5.85^{a}	19.1 ± 3.8^{a}	13.9 ± 1.28^{c}	22.7 ± 1.24^{b}	26 ± 1.32^{a}
**22:3n-3**	0 ± 0^{a}	3.29 ± 3.29^{a}	0 ± 0^{a}				0 ± 0^{a}	2.74 ± 1.86^{a}	3.99 ± 2.04^{a}
**22:5n-3**	4.24 ± 0.96^{a}	5.88 ± 0.22^{a}	5.77 ± 0.61^{a}	8.73 ± 3.38^{a}	7.08 ± 1.24^{a}	4.82 ± 0.69^{a}	5.33 ± 0.65^{b}	6.46 ± 0.6^{a}	7.36 ± 0.57^{a}
**22:6n-3**	18.5 ± 6.26^{a}	27.5 ± 4.93^{a}	23.8 ± 5.87^{a}	7.83 ± 2.06^{b}	13.4 ± 2.28^{ab}	15.1 ± 3.56^{a}	6.18 ± 0.69^{b}	9.55 ± 0.56^{a}	10.3 ± 0.68^{a}
**24:5n-3**	0 ± 0^{a}	0.1 ± 0.08^{a}	0.01 ± 0.01^{a}	0 ± 0^{a}	0.02 ± 0.02^{a}	0.03 ± 0.02^{a}	0 ± 0^{a}	0 ± 0^{a}	0.03 ± 0.03^{a}
**24:6n-3**	0.05 ± 0.04^{a}	0.11 ± 0^{a}	0.09 ± 0.02^{a}	0 ± 0^{b}	0.05 ± 0.03^{ab}	0.09 ± 0.03^{a}	0.03 ± 0.03^{a}	0 ± 0^{a}	0.02 ± 0.01^{a}
**16:2n-4**	1.19 ± 0.4^{a}	0.03 ± 0.03^{a}	1.26 ± 0.63^{a}	1.92 ± 0.57^{a}	0.56 ± 0.56^{ab}	0.27 ± 0.27^{b}	1.78 ± 0.18^{a}	1.39 ± 0.49^{ab}	0.57 ± 0.37^{b}
**16:3n-4**	1.17 ± 0.73^{a}	3.14 ± 0.72^{a}	2.73 ± 1.24^{a}	0 ± 0^{b}	0.73 ± 0.42^{ab}	1.81 ± 0.84^{a}	0.04 ± 0.04^{b}	0.18 ± 0.12^{ab}	1.23 ± 0.65^{a}
**18:2n-6**	7.17 ± 2.28^{a}	9.42 ± 1.51^{a}	7.78 ± 1.75^{a}	2.63 ± 0.58^{a}	2.63 ± 0.41^{a}	3.91 ± 1.07^{a}	2.64 ± 0.22^{ab}	2.69 ± 0.24^{b}	3.68 ± 0.48^{a}
**18:2n-6t**	0.35 ± 0.07^{a}	0.01 ± 0.01^{c}	0.05 ± 0.01^{b}	0 ± 0^{a}	0 ± 0^{a}	0.04 ± 0.02^{a}	0.08 ± 0.06^{a}	0 ± 0^{a}	0.06 ± 0.05^{a}
**18:3n-6**	29.6 ± 11.5^{a}	43.3 ± 9.99^{a}	37.7 ± 12.2^{a}	8.44 ± 3.03^{a}	14.4 ± 2.6^{a}	19.3 ± 5.71^{a}	7.94 ± 0.89^{c}	10.5 ± 0.75^{b}	18.7 ± 3.05^{a}
**20:2n-6**	1.71 ± 0.37^{a}	1.85 ± 0.13^{a}	1.74 ± 0.18^{a}	0.89 ± 0.14^{a}	1.22 ± 0.17^{a}	1.18 ± 0.19^{a}	1.01 ± 0.09^{b}	1.32 ± 0.09^{a}	1.18 ± 0.08^{ab}
**20:3n-6**	3.71 ± 1.13^{a}	5.2 ± 1.19^{a}	4.48 ± 1.23^{a}	1.21 ± 0.7^{a}	1.22 ± 0.53^{a}	2.67 ± 1^{a}	0.31 ± 0.17^{c}	0.89 ± 0.19^{b}	1.76 ± 0.32^{a}
**20:4n-6**	12.8 ± 1.9^{a}	13.5 ± 2.07^{a}	15.1 ± 3.24^{a}	16.1 ± 3.85^{a}	19.2 ± 2.37^{a}	10.8 ± 1.79^{a}	18 ± 1.75^{b}	27.2 ± 1.75^{a}	26.7 ± 1.07^{a}
**22:2n-6**	0.03 ± 0.03^{a}	0.04 ± 0.02^{a}	0.03 ± 0^{a}	0 ± 0^{b}	0 ± 0^{b}	0.05 ± 0.03^{a}	0.05 ± 0.04^{a}	0.02 ± 0.02^{a}	0.01 ± 0.01^{a}
**22:4n-6**	6.06 ± 1.01^{a}	4.42 ± 2.31^{a}	8.75 ± 2.17^{a}	7.89 ± 1.68^{a}	9.4 ± 1.23^{a}	6.86 ± 1.37^{a}	7.28 ± 0.92^{a}	10.2 ± 1.63^{a}	8.24 ± 1.88^{a}
**22:5n-6**	0.14 ± 0.14^{a}	0 ± 0^{a}	0.02 ± 0.02^{a}				0.02 ± 0.02^{a}	0 ± 0^{a}	0.03 ± 0.02^{a}
**Total**	380 ± 115^{a}	495 ± 81.4^{a}	454 ± 91.2^{a}	261 ± 49.9^{a}	306 ± 40.1^{a}	364 ± 71.2^{a}	224 ± 19.8^{b}	275 ± 18.4^{a}	339 ± 29.2^{a}
**∑SFA**	199 ± 60.2^{a}	272 ± 43.1^{a}	246 ± 57.2^{a}	158 ± 36^{a}	163 ± 25.4^{a}	222 ± 55.4^{a}	126 ± 13.6^{b}	130 ± 11.9^{b}	174 ± 19.5^{a}
**∑MUFA**	72.6 ± 25.3^{a}	70.5 ± 23.6^{a}	66.4 ± 14.9^{a}	24 ± 6.94^{b}	35.9 ± 7^{ab}	50.2 ± 4.82^{a}	29.3 ± 2.37^{b}	42.7 ± 10.6^{b}	48.2 ± 6.19^{a}
**∑PUFA**	108 ± 30.3^{a}	152 ± 18^{a}	142 ± 19.7^{a}	78.9 ± 11.3^{a}	107 ± 14.3^{a}	92.7 ± 17.2^{a}	68.8 ± 5.82^{b}	103 ± 3.92^{a}	117 ± 6.42^{a}
**∑TRANS**	0.39 ± 0.05^{a}	0.29 ± 0.09^{a}	1.06 ± 0.43^{a}	0 ± 0^{b}	0.18 ± 0.12^{ab}	1.66 ± 1.13^{a}	0.2 ± 0.11^{a}	4.61 ± 2.98^{a}	0.38 ± 0.18^{a}

**∑n-3 PUFA**	44.4 ± 12.7^{a}	70.9 ± 8.06^{a}	61.9 ± 7.4^{a}	39.9 ± 6.65^{a}	57.6 ± 10.3^{a}	45.7 ± 9.06^{a}	29.7 ± 2.74^{b}	48.2 ± 2.42^{a}	54.8 ± 3.58^{a}
**∑n-3 LC PUFA**	39.1 ± 11^{a}	61.7 ± 8.09^{a}	54 ± 6.05^{a}	35.1 ± 6.31^{a}	49.6 ± 8.68^{a}	39.5 ± 7.83^{a}	25.6 ± 2.23^{b}	41.6 ± 2.16^{a}	48.1 ± 3.27^{a}
**∑n-6 PUFA**	61.5 ± 16.7^{a}	77.7 ± 11^{a}	75.7 ± 10.6^{a}	37.1 ± 5.73^{a}	48.1 ± 4.37^{a}	44.8 ± 7.47^{a}	37.3 ± 3.21^{b}	52.8 ± 2.86^{a}	60.4 ± 3.84^{a}
**∑n-6 LC PUFA**	22.7 ± 3.83^{a}	23.1 ± 0.61^{a}	28.4 ± 4.33^{a}	25.2 ± 5.5^{a}	29.8 ± 3.32^{a}	20.4 ± 2.5^{a}	25.6 ± 2.54^{b}	38.3 ± 2.75^{a}	36.8 ± 2.11^{a}
**EPA:DHA**	1.01 ± 0.16^{a}	0.94 ± 0.17^{a}	1.18 ± 0.36^{a}	2.81 ± 0.66^{a}	2.21 ± 0.3^{ab}	1.36 ± 0.23^{b}	2.38 ± 0.23^{a}	2.4 ± 0.1^{a}	2.57 ± 0.11^{a}
**EPA:ARA**	1.19 ± 0.2^{a}	1.83 ± 0.18^{a}	1.76 ± 0.43^{a}	1.28 ± 0.19^{a}	1.51 ± 0.23^{a}	1.83 ± 0.28^{a}	0.79 ± 0.04^{b}	0.86 ± 0.06^{ab}	0.98 ± 0.05^{a}
**n-3:n-6**	0.71 ± 0.02^{b}	0.93 ± 0.09^{a}	0.82 ± 0.02^{a}	1.12 ± 0.16^{a}	1.17 ± 0.12^{a}	1.01 ± 0.03^{a}	0.81 ± 0.04^{a}	0.94 ± 0.06^{a}	0.93 ± 0.07^{a}

Supplementary Table S6. Comparison of fatty acid composition (mg g lipid^{-1}) of sample types between locations. Values are presented as means ± SEM. Values in the same row and group (i.e. sample type) that do not share the same superscripts are significantly different ($p < 0.05$).

	**HH**			**HD**			**DD**		
**Fatty acid**	**GBR**	**Palmyra**	**WA**	**GBR**	**Palmyra**	**WA**	**GBR**	**Palmyra**	**WA**
**6:0**	0.13 ± 0.03^{a}	0.09 ± 0.05^{a}	0.02 ± 0.02^{a}	0.14 ± 0.05^{a}	0.16 ± 0.1^{a}	0.06 ± 0.04^{a}	0.39 ± 0.12^{a}	0.13 ± 0.13^{a}	0.15 ± 0.07^{a}
**8:0**				0 ± 0^{b}	0 ± 0^{b}	0.03 ± 0.02^{a}	0 ± 0^{a}	0 ± 0^{a}	0.01 ± 0.01^{a}
**10:0**	2.14 ± 0.94^{a}	0.03 ± 0.02^{a}	0.14 ± 0.04^{a}	0.06 ± 0.03^{b}	0.02 ± 0.02^{b}	0.27 ± 0.12^{a}	0.01 ± 0.01^{b}	0 ± 0^{b}	0.07 ± 0.03^{a}
**11:0**	0.02 ± 0.02^{a}	0.01 ± 0.01^{a}	0.04 ± 0.02^{a}	0 ± 0^{a}	0.02 ± 0.02^{a}	0 ± 0^{a}	0 ± 0^{a}	0 ± 0^{a}	0.01 ± 0.01^{a}
**12:0**	1.04 ± 0.13^{a}	0.32 ± 0.04^{b}	0.77 ± 0.16^{a}	0.88 ± 0.12^{a}	0.48 ± 0.05^{b}	0.55 ± 0.03^{ab}	1.05 ± 0.26^{a}	0.34 ± 0.2^{a}	0.56 ± 0.16^{a}
**13:0**	0.26 ± 0.07^{a}	0.26 ± 0.11^{a}	0.16 ± 0.09^{a}	0.25 ± 0.03^{ab}	0.32 ± 0.07^{a}	0.16 ± 0.03^{b}	0.08 ± 0.04^{a}	0.13 ± 0.07^{a}	0.21 ± 0.05^{a}
**14:0**	12.8 ± 2.15^{a}	18.6 ± 5.14^{a}	16.6 ± 4.28^{a}	8.88 ± 0.87^{b}	14.7 ± 2.39^{a}	19.9 ± 2.18^{a}	7.39 ± 0.72^{a}	10.1 ± 3.86^{a}	14 ± 3.95^{a}
**15:0**	0.39 ± 0.04^{a}	0.35 ± 0.17^{ab}	0.15 ± 0.03^{b}	0.4 ± 0.05^{a}	0.31 ± 0.05^{a}	0.22 ± 0.01^{a}	0.64 ± 0.09^{a}	0.25 ± 0.11^{b}	0.34 ± 0.1^{ab}
**16:0**	121 ± 15.6^{a}	165 ± 44.1^{a}	201 ± 53^{a}	82.1 ± 9.97^{b}	114 ± 19.9^{ab}	221 ± 45^{a}	76.2 ± 7.47^{a}	97.7 ± 26.3^{a}	160 ± 53.4^{a}
**17:0**	0.44 ± 0.13^{a}	0.44 ± 0.23^{a}	0.46 ± 0.16^{a}	0.56 ± 0.12^{a}	0.31 ± 0.09^{a}	0.52 ± 0.14^{a}	1.68 ± 0.31^{a}	1.83 ± 1.03^{ab}	0.28 ± 0.14^{b}
**18:0**	27.9 ± 1.23^{a}	30.3 ± 5.89^{a}	22.3 ± 3.31^{a}	28.2 ± 1.5^{a}	25.7 ± 3.42^{a}	23.6 ± 3.88^{a}	25.3 ± 3.71^{a}	32.3 ± 9.23^{a}	17.7 ± 3.61^{a}
**20:0**	3.82 ± 0.19^{a}	4.36 ± 0.94^{a}	3.2 ± 0.42^{a}	3.66 ± 0.2^{a}	3.54 ± 0.48^{a}	3.7 ± 0.22^{a}	3.91 ± 0.33^{a}	3.62 ± 0.88^{a}	3.12 ± 0.68^{a}
**21:0**	0.39 ± 0.05^{a}	0.27 ± 0.06^{ab}	0.18 ± 0.05^{b}	0.54 ± 0.06^{a}	0.37 ± 0.04^{ab}	0.22 ± 0.06^{b}	0.99 ± 0.17^{a}	1.16 ± 0.63^{a}	0.48 ± 0.22^{a}
**22:0**	2.34 ± 0.43^{a}	1.38 ± 0.52^{a}	1.16 ± 0.34^{a}	2.8 ± 0.34^{a}	1.95 ± 0.15^{ab}	1.25 ± 0.46^{b}	5.69 ± 1.11^{a}	7.39 ± 4^{a}	1.45 ± 0.33^{b}
**24:0**	0.88 ± 0.16^{a}	0.56 ± 0.26^{a}	0.37 ± 0.11^{a}	1.25 ± 0.2^{a}	0.72 ± 0.12^{ab}	0.43 ± 0.11^{b}	2.81 ± 0.66^{a}	3.26 ± 1.66^{a}	0.64 ± 0.13^{b}

**14:1n-5**	1.43 ± 0.33^{a}	0.26 ± 0.09^{b}	0.41 ± 0.36^{b}	1.77 ± 0.34^{a}	0.83 ± 0.24^{a}	0.66 ± 0.29^{a}	1.64 ± 0.6^{a}	0.4 ± 0.27^{a}	0.4 ± 0.19^{a}
**15:1n-5**	1.05 ± 0.25^{a}	0.32 ± 0.15^{a}	0.67 ± 0.1^{a}	1.08 ± 0.23^{a}	0.56 ± 0.07^{a}	0.68 ± 0.14^{a}	1 ± 0.35^{a}	0.18 ± 0.11^{a}	0.48 ± 0.12^{a}
**16:1n-7**	5.78 ± 1.32^{a}	6.29 ± 1.56^{a}	16.6 ± 5.8^{a}	3.3 ± 0.28^{b}	4.65 ± 0.61^{a}	19.3 ± 6.86^{a}	3.05 ± 0.25^{b}	2.82 ± 1.08^{b}	14.6 ± 6.47^{a}
**17:1n-7**	17.9 ± 5.19^{a}	19.4 ± 8.83^{a}	3.99 ± 3.99^{a}	11.6 ± 1.9^{a}	17.6 ± 4.67^{a}	10.9 ± 10.9^{a}	8.62 ± 0.94^{ab}	6.88 ± 5.2^{b}	23 ± 6.88^{a}
**18:1n-7**	0.61 ± 0.08^{a}	2.41 ± 1.38^{a}	0.98 ± 0.29^{a}	2.07 ± 1.48^{a}	0.46 ± 0.06^{a}	1.09 ± 0.31^{a}	1.4 ± 0.27^{a}	0.32 ± 0.11^{b}	1.25 ± 0.3^{a}
**18:1n-7t**	0.1 ± 0.06^{b}	0.19 ± 0.12^{ab}	0.5 ± 0.2^{a}	0.05 ± 0.05^{a}	0.07 ± 0.04^{a}	0.04 ± 0.04^{a}	0.01 ± 0.01^{a}	0 ± 0^{a}	0.03 ± 0.02^{a}
**18:1n-9**	14.4 ± 2.71^{a}	13.8 ± 2.38^{a}	31.3 ± 10.2^{a}	13 ± 5.89^{b}	6.89 ± 1.13^{b}	31 ± 10.5^{a}	7.71 ± 0.55^{a}	6.82 ± 1.31^{a}	23.6 ± 9.22^{a}
**18:1n-9t**	0.22 ± 0.13^{a}	1.43 ± 1.16^{a}	0.51 ± 0.24^{a}	4.57 ± 2.98^{a}	0.11 ± 0.09^{a}	0.24 ± 0.11^{a}	0.11 ± 0.11^{a}	0 ± 0^{a}	0.01 ± 0.01^{a}
**20:1n-9**	5.57 ± 0.59^{ab}	4.48 ± 1.45^{b}	9.8 ± 2.13^{a}	3.82 ± 0.4^{a}	3.05 ± 0.61^{a}	5.45 ± 3.63^{a}	2.93 ± 0.27^{ab}	2.12 ± 0.43^{b}	7.81 ± 2.51^{a}
**22:1n-9**	0.96 ± 0.34^{a}	1.43 ± 0.66^{a}	0.72 ± 0.22^{a}	1.2 ± 0.28^{a}	1.53 ± 0.33^{a}	0.38 ± 0.03^{b}	2.56 ± 0.62^{a}	4.37 ± 1.99^{a}	0.52 ± 0.11^{b}
**24:1n-9**	0.07 ± 0.03^{a}	0.05 ± 0.04^{a}	0.11 ± 0.05^{a}	0.01 ± 0.01^{b}	0.08 ± 0.06^{ab}	0.07 ± 0.02^{a}	0.04 ± 0.03^{b}	0 ± 0^{b}	0.18 ± 0.12^{a}
**20:1n-11**	0 ± 0^{b}	0 ± 0^{b}	0.64 ± 0.22^{a}	0 ± 0^{b}	0 ± 0^{b}	0.48 ± 0.27^{a}	0 ± 0^{b}	0 ± 0^{b}	0.46 ± 0.25^{a}
**22:1n-11**	0.13 ± 0.02^{a}	0.09 ± 0.03^{a}	0.15 ± 0.03^{a}	0.24 ± 0.14^{a}	0.12 ± 0.06^{a}	0.15 ± 0.04^{a}	0.28 ± 0.1^{a}	0.13 ± 0.09^{a}	0.23 ± 0.07^{a}
**18:3n-3**	0.1 ± 0.03^{a}	0.06 ± 0.04^{a}	0.13 ± 0.02^{a}	0.16 ± 0.09^{a}	0.02 ± 0.02^{a}	0.15 ± 0.04^{a}	0.31 ± 0.12^{a}	0.02 ± 0.02^{ab}	0.21 ± 0.05^{a}
**18:4n-3**	6.66 ± 0.61^{a}	6.14 ± 1.27^{a}	7.84 ± 1.34^{a}	6.43 ± 0.7^{a}	7.99 ± 1.67^{a}	9.04 ± 0.84^{a}	3.8 ± 0.46^{a}	4.71 ± 0.37^{a}	5.14 ± 1.83^{a}
**20:4n-3**	0.42 ± 0.1^{a}	0.43 ± 0.25^{a}	0.46 ± 0.23^{a}	0.22 ± 0.08^{b}	0.12 ± 0.12^{b}	0.7 ± 0.16^{a}	0.08 ± 0.05^{b}	0 ± 0^{b}	0.44 ± 0.17^{a}
**20:5n-3**	26 ± 1.32^{a}	19.1 ± 3.8^{a}	23.8 ± 0.68^{a}	22.7 ± 1.24^{a}	29 ± 5.85^{a}	24.2 ± 2.73^{a}	13.9 ± 1.28^{a}	18.6 ± 3.17^{a}	15.8 ± 3.82^{a}
**22:3n-3**	3.99 ± 2.04^{a}	0 ± 0^{a}	0 ± 0^{a}	2.74 ± 1.86^{a}	0 ± 0^{a}	3.29 ± 3.29^{a}			
**22:5n-3**	7.36 ± 0.57^{a}	4.82 ± 0.69^{b}	5.77 ± 0.61^{ab}	6.46 ± 0.6^{a}	7.08 ± 1.24^{a}	5.88 ± 0.22^{a}	5.33 ± 0.65^{a}	8.73 ± 3.38^{a}	4.24 ± 0.96^{a}
**22:6n-3**	10.3 ± 0.68^{b}	15.1 ± 3.56^{ab}	23.8 ± 5.87^{a}	9.55 ± 0.56^{b}	13.4 ± 2.28^{ab}	27.5 ± 4.93^{a}	6.18 ± 0.69^{a}	7.83 ± 2.06^{a}	18.5 ± 6.26^{a}
**24:5n-3**	0.03 ± 0.03^{a}	0.03 ± 0.02^{a}	0.01 ± 0.01^{a}	0 ± 0^{b}	0.02 ± 0.02^{ab}	0.1 ± 0.08^{a}			
**24:6n-3**	0.02 ± 0.01^{b}	0.09 ± 0.03^{a}	0.09 ± 0.02^{a}	0 ± 0^{c}	0.05 ± 0.03^{b}	0.11 ± 0^{a}	0.03 ± 0.03^{a}	0 ± 0^{a}	0.05 ± 0.04^{a}
**16:2n-4**	0.57 ± 0.37^{a}	0.27 ± 0.27^{a}	1.26 ± 0.63^{a}	1.39 ± 0.49^{a}	0.56 ± 0.56^{a}	0.03 ± 0.03^{a}	1.78 ± 0.18^{a}	1.92 ± 0.57^{a}	1.19 ± 0.4^{a}
**16:3n-4**	1.23 ± 0.65^{a}	1.81 ± 0.84^{a}	2.73 ± 1.24^{a}	0.18 ± 0.12^{b}	0.73 ± 0.42^{b}	3.14 ± 0.72^{a}	0.04 ± 0.04^{b}	0 ± 0^{b}	1.17 ± 0.73^{a}
**18:2n-6**	3.68 ± 0.48^{b}	3.91 ± 1.07^{ab}	7.78 ± 1.75^{a}	2.69 ± 0.24^{b}	2.63 ± 0.41^{b}	9.42 ± 1.51^{a}	2.64 ± 0.22^{a}	2.63 ± 0.58^{a}	7.17 ± 2.28^{a}
**18:2n-6t**	0.06 ± 0.05^{a}	0.04 ± 0.02^{a}	0.05 ± 0.01^{a}	0 ± 0^{b}	0 ± 0^{b}	0.01 ± 0.01^{a}	0.08 ± 0.06^{b}	0 ± 0^{b}	0.35 ± 0.07^{a}
**18:3n-6**	18.7 ± 3.05^{a}	19.3 ± 5.71^{a}	37.7 ± 12.2^{a}	10.5 ± 0.75^{b}	14.4 ± 2.6^{ab}	43.3 ± 9.99^{a}	7.94 ± 0.89^{a}	8.44 ± 3.03^{a}	29.6 ± 11.5^{a}
**20:2n-6**	1.18 ± 0.08^{b}	1.18 ± 0.19^{b}	1.74 ± 0.18^{a}	1.32 ± 0.09^{b}	1.22 ± 0.17^{b}	1.85 ± 0.13^{a}	1.01 ± 0.09^{a}	0.89 ± 0.14^{a}	1.71 ± 0.37^{a}
**20:3n-6**	1.76 ± 0.32^{a}	2.67 ± 1^{a}	4.48 ± 1.23^{a}	0.89 ± 0.19^{b}	1.22 ± 0.53^{b}	5.2 ± 1.19^{a}	0.31 ± 0.17^{b}	1.21 ± 0.7^{b}	3.71 ± 1.13^{a}
**20:4n-6**	26.7 ± 1.07^{a}	10.8 ± 1.79^{b}	15.1 ± 3.24^{b}	27.2 ± 1.75^{a}	19.2 ± 2.37^{b}	13.5 ± 2.07^{b}	18 ± 1.75^{a}	16.1 ± 3.85^{a}	12.8 ± 1.9^{a}

**22:2n-6**	0.01 ± 0.01^{b}	0.05 ± 0.03^{a}	0.03 ± 0^{a}	0.02 ± 0.02^{a}	0 ± 0^{a}	0.04 ± 0.02^{a}	0.05 ± 0.04^{a}	0 ± 0^{a}	0.03 ± 0.03^{a}
**22:4n-6**	8.24 ± 1.88^{a}	6.86 ± 1.37^{a}	8.75 ± 2.17^{a}	10.2 ± 1.63^{a}	9.4 ± 1.23^{ab}	4.42 ± 2.31^{b}	7.28 ± 0.92^{a}	7.89 ± 1.68^{a}	6.06 ± 1.01^{a}
**22:5n-6**	0.03 ± 0.02^{a}	0 ± 0^{a}	0.02 ± 0.02^{a}				0.02 ± 0.02^{b}	0 ± 0^{b}	0.14 ± 0.14^{a}
**Total**	339 ± 29.2^{a}	364 ± 71.2^{a}	454 ± 91.2^{a}	275 ± 18.4^{b}	306 ± 40.1^{ab}	495 ± 81.4^{a}	224 ± 19.8^{a}	261 ± 49.9^{a}	380 ± 115^{a}
**∑SFA**	174 ± 19.5^{a}	222 ± 55.4^{a}	246 ± 57.2^{a}	130 ± 11.9^{b}	163 ± 25.4^{ab}	272 ± 43.1^{a}	126 ± 13.6^{a}	158 ± 36^{a}	199 ± 60.2^{a}
**∑MUFA**	48.2 ± 6.19^{a}	50.2 ± 4.82^{a}	66.4 ± 14.9^{a}	42.7 ± 10.6^{a}	35.9 ± 7^{a}	70.5 ± 23.6^{a}	29.3 ± 2.37^{a}	24 ± 6.94^{a}	72.6 ± 25.3^{a}
**∑PUFA**	117 ± 6.42^{ab}	92.7 ± 17.2^{b}	142 ± 19.7^{a}	103 ± 3.92^{b}	107 ± 14.3^{ab}	152 ± 18^{a}	68.8 ± 5.82^{a}	78.9 ± 11.3^{a}	108 ± 30.3^{a}
**∑TRANS**	0.38 ± 0.18^{a}	1.66 ± 1.13^{a}	1.06 ± 0.43^{a}	4.61 ± 2.98^{a}	0.18 ± 0.12^{a}	0.29 ± 0.09^{a}	0.2 ± 0.11^{b}	0 ± 0^{b}	0.39 ± 0.05^{a}
**∑n-3 PUFA**	54.8 ± 3.58^{a}	45.7 ± 9.06^{a}	61.9 ± 7.4^{a}	48.2 ± 2.42^{b}	57.6 ± 10.3^{ab}	70.9 ± 8.06^{a}	29.7 ± 2.74^{a}	39.9 ± 6.65^{a}	44.4 ± 12.7^{a}
**∑n-3 LC PUFA**	48.1 ± 3.27^{a}	39.5 ± 7.83^{a}	54 ± 6.05^{a}	41.6 ± 2.16^{b}	49.6 ± 8.68^{ab}	61.7 ± 8.09^{a}	25.6 ± 2.23^{a}	35.1 ± 6.31^{a}	39.1 ± 11^{a}
**∑n-6 PUFA**	60.4 ± 3.84^{ab}	44.8 ± 7.47^{b}	75.7 ± 10.6^{a}	52.8 ± 2.86^{b}	48.1 ± 4.37^{b}	77.7 ± 11^{a}	37.3 ± 3.21^{a}	37.1 ± 5.73^{a}	61.5 ± 16.7^{a}
**∑n-6 LC PUFA**	36.8 ± 2.11^{a}	20.4 ± 2.5^{b}	28.4 ± 4.33^{b}	38.3 ± 2.75^{a}	29.8 ± 3.32^{ab}	23.1 ± 0.61^{b}	25.6 ± 2.54^{a}	25.2 ± 5.5^{a}	22.7 ± 3.83^{a}
**EPA:DHA**	2.57 ± 0.11^{a}	1.36 ± 0.23^{b}	1.18 ± 0.36^{b}	2.4 ± 0.1^{a}	2.21 ± 0.3^{a}	0.94 ± 0.17^{b}	2.38 ± 0.23^{a}	2.81 ± 0.66^{a}	1.01 ± 0.16^{b}
**EPA:ARA**	0.98 ± 0.05^{b}	1.83 ± 0.28^{a}	1.76 ± 0.43^{a}	0.86 ± 0.06^{b}	1.51 ± 0.23^{a}	1.83 ± 0.18^{a}	0.79 ± 0.04^{b}	1.28 ± 0.19^{a}	1.19 ± 0.2^{a}
**n-3:n-6**	0.93 ± 0.07^{a}	1.01 ± 0.03^{a}	0.82 ± 0.02^{a}	0.94 ± 0.06^{a}	1.17 ± 0.12^{a}	0.93 ± 0.09^{a}	0.81 ± 0.04^{ab}	1.12 ± 0.16^{a}	0.71 ± 0.02^{b}

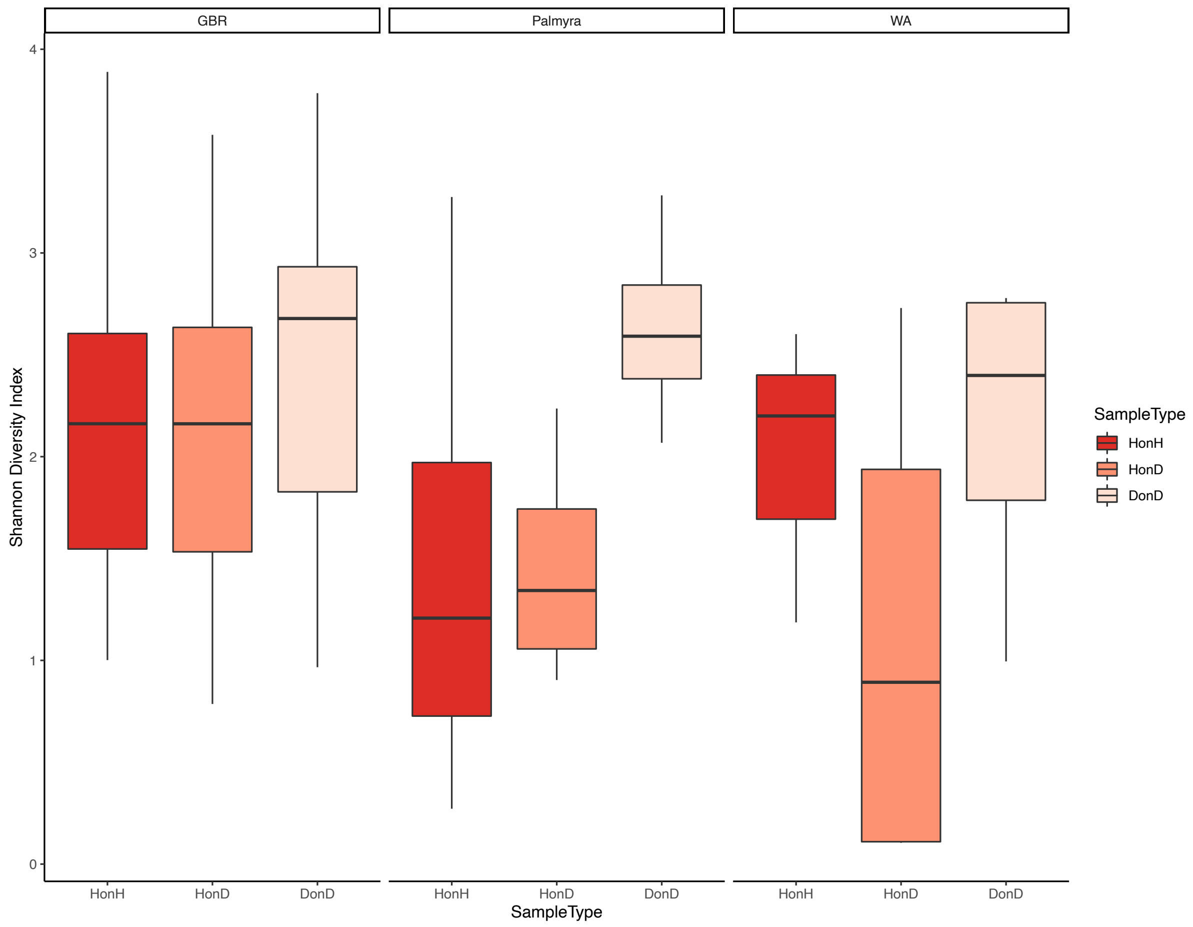

Supplementary Figure S3. Shannon diversity index for microbial communities associated with healthy and diseased colonies of *A hyacinthus*.

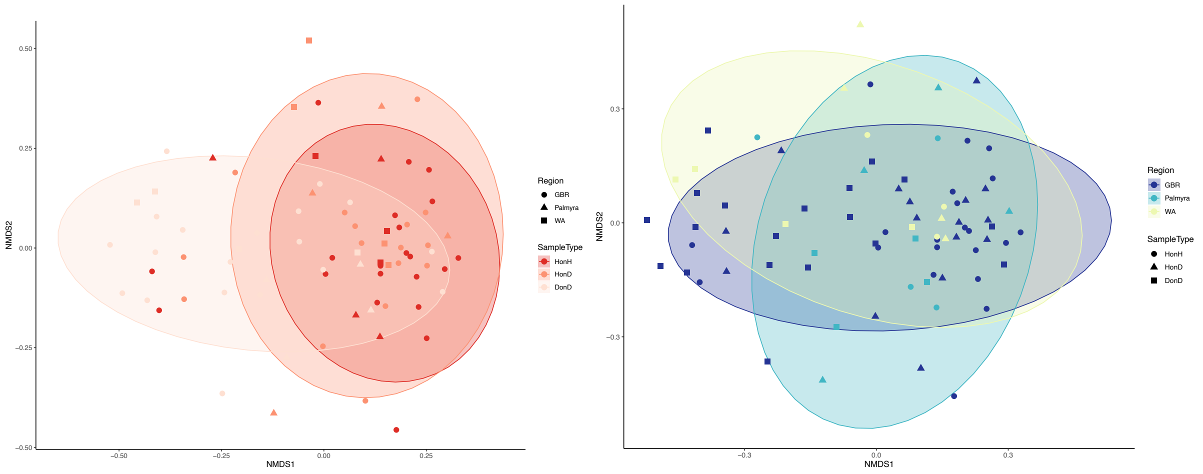

Supplementary Figure S4. nMDS of microbial community composition with ellipses representing 90% confidence intervals for A) health states and B) locations.

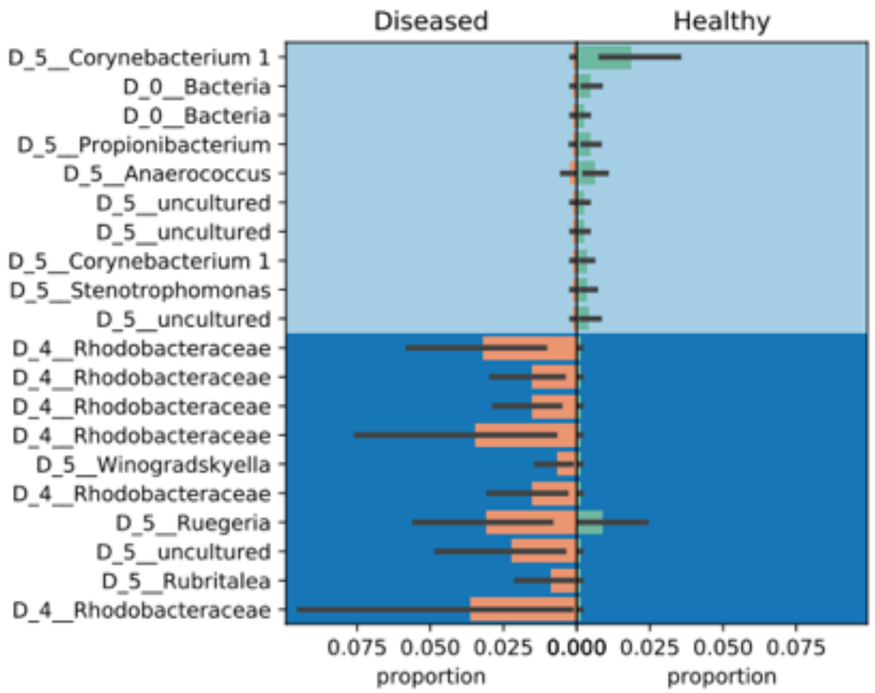

Supplementary Figure S5. Correlation clustering identified sequence variants with significantly different relative abundance in diseased and healthy tissue.
